# Computational modelling of nanotube delivery of anti-cancer drug into glutathione reductase enzyme

**DOI:** 10.1038/s41598-021-84006-1

**Published:** 2021-03-02

**Authors:** Saheen Shehnaz Begum, Dharitri Das, Nand Kishor Gour, Ramesh Chandra Deka

**Affiliations:** Department of Chemical Sciences, Tezpur University, Tezpur, Assam 784028 India

**Keywords:** Biophysics, Cancer, Computational biology and bioinformatics, Molecular medicine, Chemistry, Nanoscience and technology

## Abstract

Density functional theory method combined with docking and molecular dynamics simulations are used to understand the interaction of carmustine with human glutathione reductase enzyme. The active site of the enzyme is evaluated by docking simulation is used for molecular dynamics simulation to deliver the carmustine molecule by (5,5) single walled carbon nanotube (SWCNT). Our model of carmustine in the active site of GR gives a negative binding energy that is further refined by QM/MM study in gas phase and solvent phase to confirm the stability of the drug molecule inside the active site. Once released from SWCNT, carmustine forms multiple polar and non-polar hydrogen bonding interactions with Tyr180, Phe209, Lys318, Ala319, Leu320, Leu321, Ile350, Thr352 and Val354 in the range of 2–4 Å. The SWCNT vehicle itself is held fix at its place due to multiple pi-pi stacking, pi-amide, pi-sigma interactions with the neighboring residues. These interactions in the range of 3–5 Å are crucial in holding the nanotube outside the drug binding region, hence, making an effective delivery. This study can be extended to envisage the potential applications of computational studies in the modification of known drugs to find newer targets and designing new and improved controlled drug delivery systems.

## Introduction

Combination of computational tools such as docking, molecular dynamics simulations as well as QM/MM are powerful and necessary to explain the biological environment that loses its essence outside the human cell. In human erythrocytes, glutathione reductase (GR) catalyzes the reduction of glutathione disulfide to glutathione which maintains the reducing environment of the cell with the primary function of maintaining a high intracellular ratio of [GSH]/GR], where GSH is reduced glutathione. The oxidative stress of the cell increases in the absence of glutathione, with the generation of peroxides and free radicals that cause damage to all cellular components. The GR family consists of a central five-stranded parallel β-sheet surrounded by α-helices and an additional crossover connection composed of a three-stranded antiparallel β-sheet^1^. It is an oxidoreductase homodimer with 52 kD monomers, of which each has three domains viz. residues 1–157 FAD-binding domain, residues 158–293 NADPH-binding domain, and residues 365–478 dimerization domain^2,3^.

Carmustine is β-chloro-nitrosourea (BCNU) is a nitrosourea whose primary function is the alkylation of DNA^4–6^. During craniotomy, a piece of bone is removed to expose the area of brain over the tumor. The outermost layer of the brain tissues (known as dura mater) is opened, thus, the tumor is located and then resected. After the tumor is removed, the bone is usually replaced and the scalp stitched shut. The removal of the tumour creates a cavity. Commercially, Gliadel wafer is a form of carmustine medication where carmustine is incorporated in the cavity after surgical removal of a brain tumor. Such carmustine wafer allows for delivery of the drug directly to the site of the brain tumor, the amount of carmustine received depends on the size of the cavity and how many wafers can be put into place^7–11^.

Alkylating agents such as carmustine, lomustine (Nitrosoureas), bendamustine and cyclophosphamide (Nitrogen mustards), busulfan (Alkyl sulfonates), temozolomide (Triazines) are some of the chemotherapeutic drug agents that are used in modern cancer chemotherapies^12^. The rationalization for using alkylating agents in chemotherapeutic cancer treatment is that such agents exert acute cytotoxic effects on actively growing cells.

However, acquired drug resistance is one of the greatest hindrances for the successful treatment of cancer. Resistance can emerge due to several reasons such as environmental factors, genetic or epigenetic alterations in the cancer cells. Hence, finding newer routes to cease actively growing cancer cells is implicit. Inhibition of specific proteins such as GR may be one of the possible routes that would lead to cancer cell death.

The role of GR enzyme inhibition by carmustine was experimentally confirmed on mice by Kehrer^13^. He determined that maximum inhibition of GR by carmustine is reached at 4 h in all tissues. This was achieved by a single 50 mg/kg ip dose of the drug and it was reported that inhibition was reached within 10 min of drug intake.

The effects of high dosage of carmustine in men and its pharmacokinetics was examined and reported by Henner et al.^14^. Doroshenko et al.^15^ elucidated the effect of carmustine on the intracellular Ca^2+^ concentration in PC12 cells. They established that 100 μM carmustine caused delayed increase in [Ca^2+^]_i_. It was concluded that carmustine prompts an influx of extracellular Ca^2+^ through L-type Ca^2+^ channels. Such an effect is mediated by oxidative stress that results from the depletion of GSH following the inhibition of glutathione reductase by carmustine.

The present work is mainly focused to examine the stability and binding affinity of carmustine with GR enzyme and shed light on the carmustine-GR interaction. In order to determine the active site, the stability and binding affinity of the anti-cancer drug with the protein receptor, two dominant computational methods have been utilized: docking and ONIOM (Our own N-layered Integrated molecular Orbital and Molecular Mechanics)^16–18^ methods. This work further utilizes molecular dynamics (MD) simulations to simulate the delivery of the drug using a SWCNT onto the receptor. Utilisation of drug delivery vehicles^19,20^ such as Single-walled carbon nanotube (SWCNT), Multi-walled carbon nanotube (MWCNT), Boron nitride nanotube (BNNT), graphene sheet–both modified and pure could be the new generation technique in treatment of diseases. The properties attributed to their unique structure as well as the electronic properties, aid in using them as molecular vehicles. Over the years several theoretical as well as experimental work^21–27^ has been done on controlled delivery of drug using nanoparticles. Significant advances have been made in manipulating the properties of the drug as well as the “vehicle” with the hope of increasing drug efficiency.

## Computational details

### Structure

GAUSSIAN 09^28^ program package is employed to carry out density functional theory (DFT) calculation to find out the optimized structure of carmustine molecule. We have used meta-GGA hybrid M062X^29–31^ functional with triple zeta split valence and polarized 6-311G(d,p) basis set^32^ for gas and solvent phase geometry optimization. M06-2X functional has been parameterized for nonmetals and is a high-nonlocality functional with double the amount of nonlocal exchange (2X), and provide satisfactory results for several structural and thermodynamic properties^33^.

### Molecular docking

DFT optimized structure of carmustine and crystal structure of human reductase obtained from research collaboratory for structural bioinformatics (RCSB) protein data bank is taken for molecular docking simulation. For carrying out this work PDB identity, 1BWC, (Structure of Human GR complexed with Ajoene inhibitor and subversive substrate) has been taken. The binding site for carmustine in the GR receptor is not known. To identify the binding site, we performed blind docking docking by setting up the dockings so as to search the entire surface of the protein (GR) by using AutoGrid to create very large grid maps, with the maximum number of points in each dimension, and if necessary, creating sets of adjacent grid map volumes that cover the macromolecule. AutoGrid is included in the AutoDock 4.2 program^34^. Once, blind docking was completed to give the most probable site for carmustine binding, we carried out docking a few more times using AutoGrid to create specific grid maps at that same site until the lowest energy docked structure was obtained.

To carry out docking, the protein structure in pdb format is prepared by structure preparation tool available in Dock Prep Tool in UCSF Chimera 1.11.2 package version^35^. All the water molecules and the residues (ajoene) have been removed from the crystal structure of GR keeping the co-factor FAD (flavin adenine dinucleotide) moiety intact. The polar hydrogen atoms are added for saturation. Gasteiger charges are computed and non-polar hydrogen atoms are merged. A grid box with grid spacing of 0.486 Å and dimension of 83 × 95 × 107 grid points along x, y and z axes is built. The grid box carries the complete protein receptor and gives sufficient space for the ligand translational and rotational walk. Finally, ten possible docking runs are performed with a maximum number of energy evaluations set to 2,500,000 and a maximum number of 27,000 GA operations. These are generated with an initial population of 150 individuals taking the rate of gene mutation and crossover set to 0.02 and 0.80, respectively.

### QM/MM

**Figure 1:**
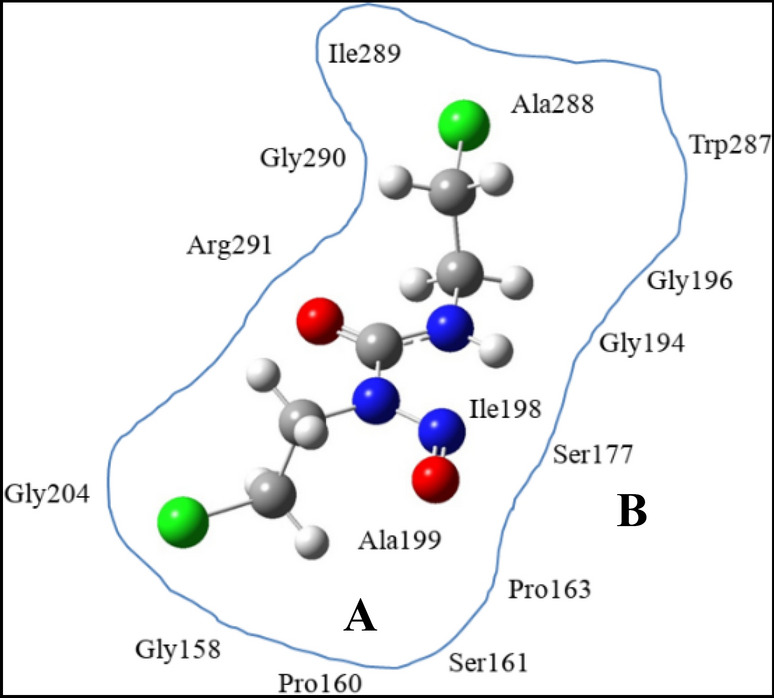
Schematic 2D diagram of the model system carmustine bound to GR binding site. Layers that are partitioned are shown for ONIOM calculations. A is the inner layer (QM calculations) and B is the outer layer (MM calculations). The arrangement of the residues is shown in 2D diagram is not their actual position in 3D.

The best docked structure is chosen as the initial geometry for the two-layer ONIOM study which is implemented within the GAUSSIAN09 suite. The system size has been reduced by removing the protein residues located outside the region of the active site of the receptor. This decreases the computational demand of investigating the whole protein–ligand adduct by quantum mechanics (QM) to a great extent without compromising the results. Thus, we have carried out QM calculation on the residues that interact with the drug and molecular mechanics (MM) for the residual part of the larger protein system (Fig. 1).

In the GR receptor protein, QM region has been constituted by Ala199, Isoleu198, Ala288, Isoleu289 and carmustine (region A in Fig. 1) whereas the MM region is composed of Trp287, Gly196, Ala195, Gly194, Pro163 Ser161, Pro163, Ser208, Arg291, Gly290, respectively (region B in Fig. 1). The charge of higher level and for molecules at lower level, is set at 0, since the moieties are neutral. Consequently, this structure is optimized using two layer ONIOM method by treating QM region at UM06-2X/6-311G (d,p) level. The MM region is described using universal force field, UFF, implemented in GAUSSIAN 09 program. With the dual layers of ONIOM method, E_ONIOM_, represents the total energy of the energy system is obtained from three independent energy evaluations.

1 $${\text{E}}^{{{\text{ONIOM}}}} = {\text{E}}^{{{\text{high}}}} \,\left( {{\text{model}}\,{\text{system}}} \right) + {\text{E}}^{{{\text{low}}}} \,\left( {{\text{real}}\,{\text{system}}} \right){-}{\text{E}}^{{{\text{low}}}} \,\left( {{\text{model}}\,{\text{system}}} \right)$$

The actual system consists of the full geometry of the molecule and is treated as MM layer while the model system consists of the chemically most important part of the system, (known as the core) of the system, which is treated as the QM layer.

The interaction energy, ∆E, has been evaluated to determine the stability of adduct which is given by the expression:

2 $$\Delta {\text{E }} = \Delta {\text{E}}_{{\text{GR/lig}}} - \Delta {\text{E}}_{{{\text{GR}}}} - \Delta {\text{E}}_{{{\text{lig}}}}$$

∆E_GR/lig_ represents the energy of the optimized adducts of the complex-GR, ∆E_GR_ represents the energy of the optimized GR receptor and the ∆E_lig_ is the energy of the optimized carmustine ligand.

The effect of solvation in the carmustine ligand-GR interaction has been observed by performing single point calculations on the interacting part of the protein by the UM06-2X functional and 6-311+G(d,p) basis set and conductor like polarized continuum model^36,37^. To reduce the computation time, only the high level (QM) has been taken for single point calculation.

### Molecular dynamics simulation

A total 100 ns MD simulations has been performed using a GROMACS package 5.1.2 with Optimized Potential for Liquid Simulations-all atom (OPLS-AA) force-field^38,39^ for each system. A (5, 5) CNT with H termination on both ends has been used as the vehicle for drug delivery (diameter of 6.78 Å and length of 9.84 Å) to accommodate the drug. At first the topology of the protein has been generated using GROMACS. After the generation of topology file for the protein, the #include option was used to include the topology information of FAD, carmustine and the SWCNT. This system was then placed in a triclinic periodic box boxed and solvated using the spc/e water model. The dipole moment of SPC/E water model is 2.35 D with a polarization correction to the total electrical energy of 5.22 kJ mol^−1^. This model, SPC/E is especially accurate for capturing diffusion coefficient, dielectric constant and other experimental properties of water and hence provide reasonable results for simulation of peptides and proteins^40^.

Post solvation 17,465 molecules of water molecules are added into the box. The total charge of the system is neutralized by adding ions after which energy minimization is carried out. The equilibration step is then carried out for 10 ns at constant pressure (1 bar) and temperature (298 K) using Berendsen pressure coupling^41^. The total energy of molecular system using OPLS-AA force field is a sum of the non-bonded energy E_nb_, bond stretching and angle bending terms E_bond_ and E_angle_, and the torsional energy E_torsion_. The non-bonded part was computed as a sum of the Coulomb and Lennard–Jones contributions for pairwise intra- and intermolecular interactions.

The results obtained after the production run of 100 ns, which generates the trajectory of the system, especially the drug released from the endohedral carbon nanotube cavity are further analyzed and plotted using xmGrace tool^42^ and the interactions have been visualized using BIOVIA Discovery Studio^43^. The change in radius of gyration (Rg) of the protein, root mean square deviation (RMSD) and root mean square fluctuations (RMSF) of the drug movement in the active site of the protein has been analyzed with respect to simulation time.

## Results and discussion

### Docking study

The analysis of molecular docking calculations was done for carmustine-GR system. The values of binding energy, inhibition constant (kI), intermolecular and internal energy of first three best docked structures for unsupported carmustine-GR system has been listed in Table 1. It is observed that the values for the different properties across the three structures do not differ significantly. The negative value of the binding energy reflects the strongly binding nature of the drug with the receptor.

**Table 1:** Docking properties for the best three docked structure.

	Binding energy	kI (uM)	Intermolecular energy	Internal energy
Rank: 1_1	− 4.7	360.98	− 6.19	− 1.12
Rank: 1_2	− 4.69	362.09	− 6.19	− 1.12
Rank: 1_3	− 4.69	365.94	− 6.18	− 1.13

Energies are in kcal mol^−1^.

**Figure 2:**
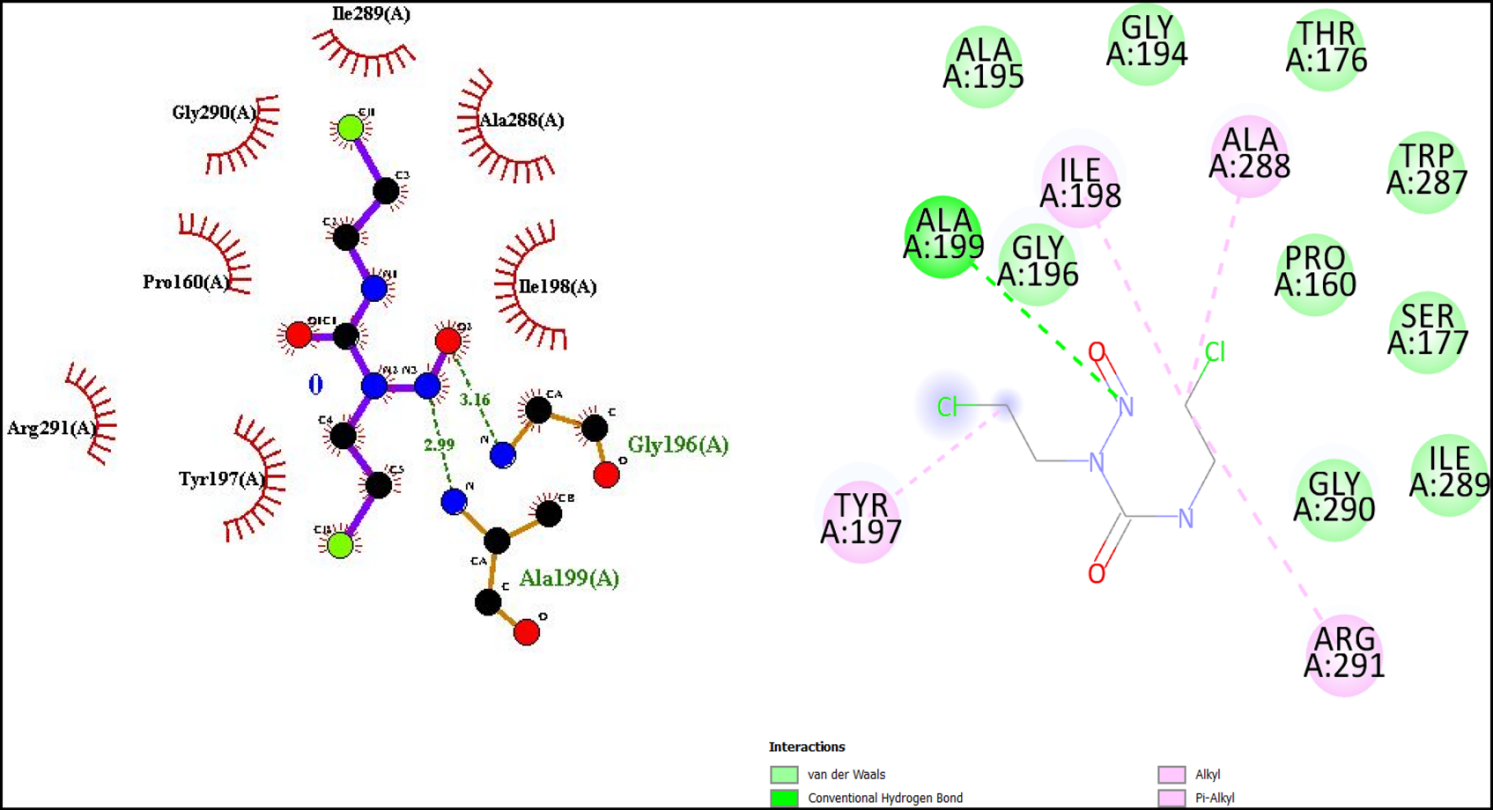
(**a**) Binding pocket of carmustine showing the hydrophobic pocket (**b**) types of carmustine-residue interactions.

For the best docked structure as calculated from AutoDock, the schematic 2-D representation of protein–ligand interactions post-docking has been generated using LigPlot^44^ program and Biovia Discovery Studio^43^ as shown in Fig. 2.

**Figure 3:**
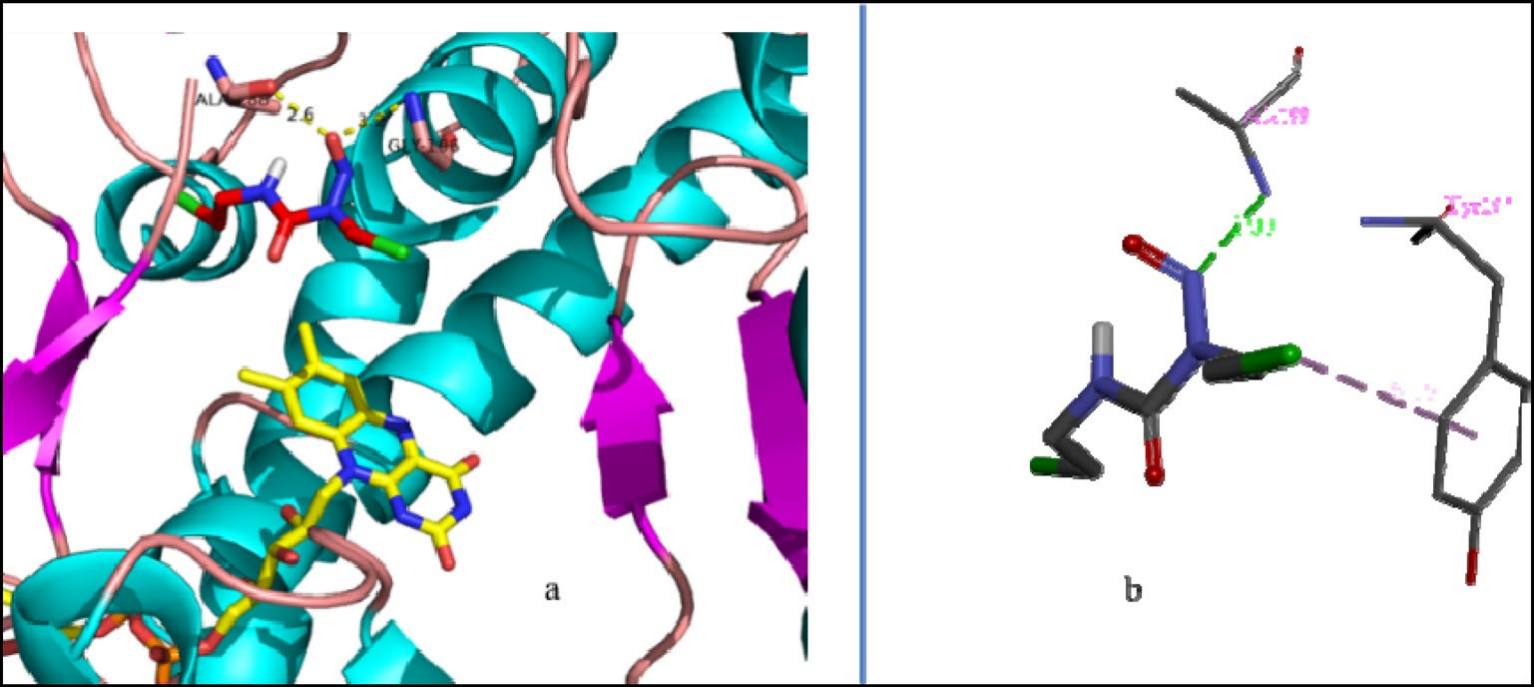
Carmustine interaction at the ligand binding site post docking calculations (**a**) At active site (**b**) interaction of carmustine with Tyr197.

The docking calculations show that carmustine binds in the NADPH binding domain of GR. Figure 2a shows the hydrophobic contacts (Pro160, Tyr197, Ile198, Ala288, Ileu289, Gly290 and Arg291) for the ligand binding show the binding pocket of carmustine in GR chain A. Carmustine forms hydrogen bonds with two residues of the active amino acids Ala199 and Gly196 at 2.99 and 3.16 Å, respectively which occur through the N-atom and O-atom of nitroso group of carmustine (Fig. 3a). The active site and the corresponding interactions of carmustine with the neighboring chain A residues is more clearly seen in Fig. 2b. The stability is due to hydrogen bonding and electrostatic interactions. Other than the conventional H-bonds, carmustine also interacts with residues in close proximity by van der Waals interactions with residues Pro160, Thr176, Ser177, Gly194, Ala195, Trp287, Ile289, Gly290. Alkyl type interactions of carmustine occurs with Ile198, Ala288 and Arg291 and pi-alkyl type interactions occurs with Tyr197.

The interaction of carmustine with Tyr197 (Fig. 3b) is particularly interesting because it is a highly conserved residue and is crucial for GR enzyme activity. In the experimental investigations of kinetic, spectroscopic and catalytic properties of human GR, it is found that mutation of Tyr197 leads to a decreased K_m_ value for Glutathione disulphide. Tyr197 serving as a gate in binding of NADPH^45^ and points almost perpendicular onto the flavin ring in an unliganded GR enzyme^46^. Binding of carmustine to Tyr197 hinders the NADPH binding to Tyr197 necessary to cause a main chain motion along with C_α_–C_β_ bond rotation, whereby the OH group is moved by 6.4 Å as reported by Karplus and Schulz^47^, which has profound effect on the catalysis process.

**Figure 4:**
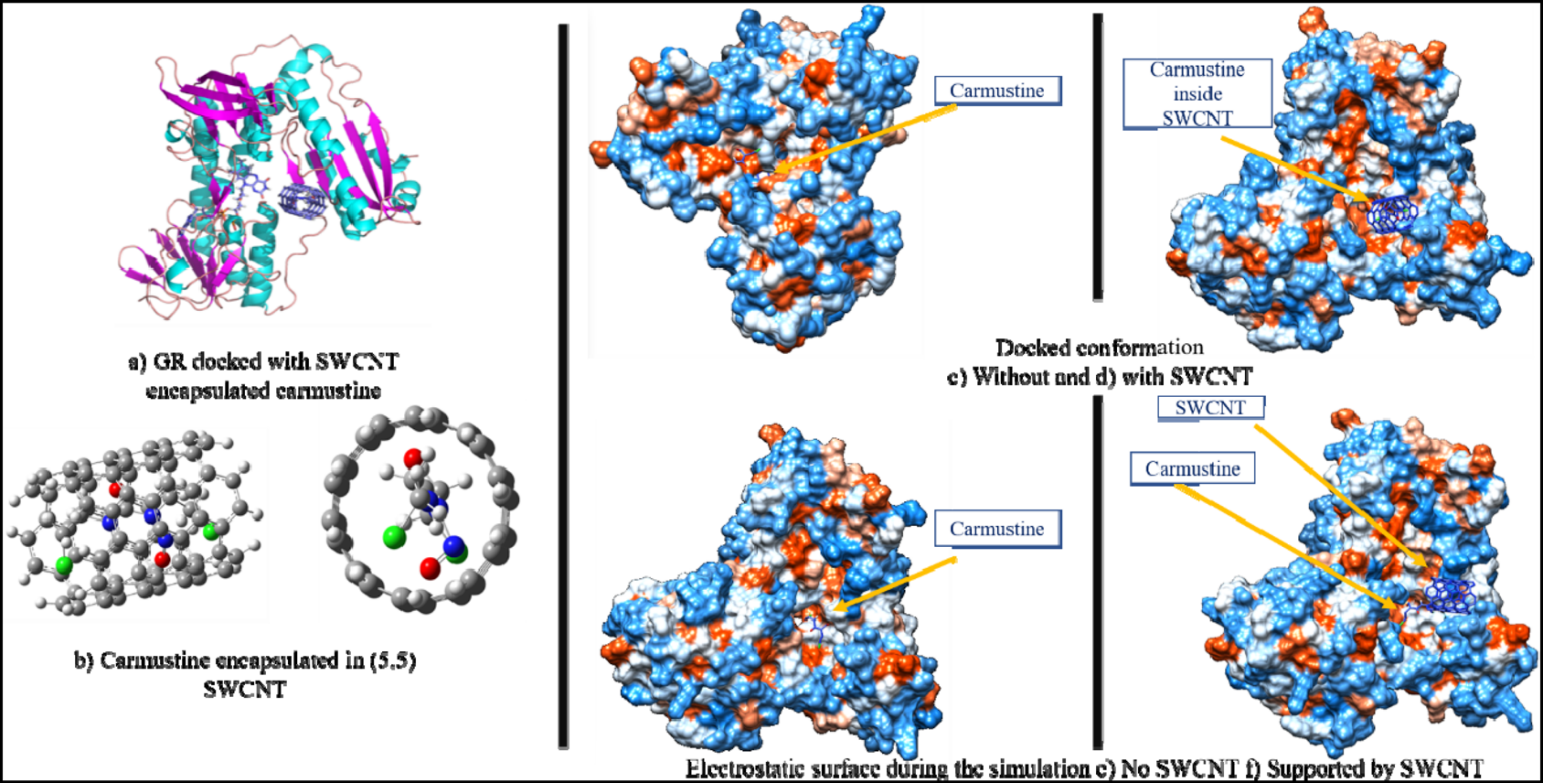
(**a**) SWCNT-carmustine docked in GR conformation (**b**) (5,5) SWCNT carrying the drug and Electrostatic surface corresponding to docking results (**c**,**d**) and during the simulation (**e**,**f**) in absence and presence of SWCNT support respectively.

To investigate the prospective role of SWCNTs in carmustine drug targeting onto a GR enzyme, we docked Carmustine encapsulated within SWCNT onto the GR enzyme defining the binding pocket within the protein as depicted in Fig. 4a,b. The electrostatic surface indicates that the carmustine molecule gets grooved within the binding pocket on GR (Fig. 4c–f).

The docking of SWCNT with carmustine has been done on GR enzyme to develop the perspective of the binding site where the nanotube will land, with our focus on simulation of release of the drug from the endohedral cavity. As seen in Table 1, carmustine shows negative values of the binding energy which reflects the high binding of the drug with the receptor that in turn suggests that the SWCNT delivered carmustine drug can sustain its physiological activity and properties. To obtain more accurate binding energy values of carmustine to the GR active site, we performed QM/MM calculations as follows.

### QM/MM study

#### Interactions and Stability

**Figure 5:**
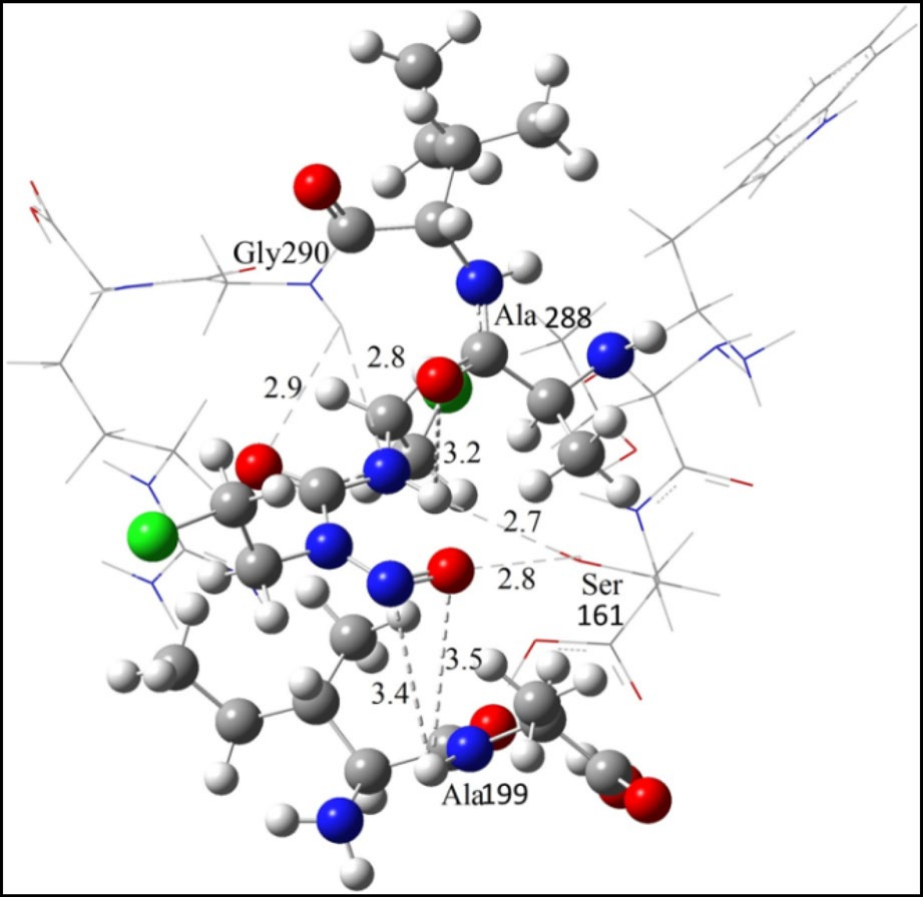
Optimized geometry of carmustine-GR adduct calculated at UM06-2X/6-311G (d,p) UFF level.

It can be seen that inside the binding site of GR, the protein retains its linearity. During QM/MM calculations, it is observed that the ligand aligns such that it forms multiple hydrogen bonding interactions with the surrounding residues (Fig. 5). Two hydrogen bonding interaction occur between N-H_Gly290_ and central N and O=C of carmustine at bond distance of 2.9 and 2.8 Å respectively, while, another two weak hydrogen bonds with N-H_Ala199_ and both atoms of N=O (of carmustine) at 3.5 and 3.4 Å respectively. A single hydrogen bonding interaction is observed with O-H_Ser161_ at 2.8 Å to O doubly bonded to N in carmustine and another H-bond at 3.2 Å with O=C_Ala288_ and N–H of carmustine.

### Binding energy

To determine the stability of carmustine-GR adduct we have evaluated the binding energy along with the absolute energy values of the molecules involved (Table 2). It is known that biological interactions always occur in aqueous environment, thus, to include the solvent effect in this system, we carried out similar calculations on the interacting regions of the protein and the ligand in the solvent medium. With the inclusion of the solvent effect the binding energy of adduct is evaluated to be higher as compared to its respective counterpart in gas phase, suggesting the increase in stability of the adduct with the inclusion of solvent. The higher binding energy in solvent medium may be due to the fact that in aqueous medium the adduct species remain in the solvation state. Solvation, solvates the species through hydrogen bonding network. The hydrogen bonding network stabilize the adduct complex to a greater extent, making the binding between drug and protein stronger and hence increases the binding as compared to gas phase adduct.

**Table 2:** Energy values (in au) of interacting adducts and calculated binding energy of carmustine ligand with GR calculated in gas phase and solvent phase.

Phase	∆E_GR/lig_	∆E_GR_	∆E_lig_	∆E (kcalmol^−1^)
Gas	− 2733.51	− 1302.56	− 1430.93	0.012 = 12.41
Solvent	− 2733.59	− 1302.62	− 1430.94	0.024 = 14.93

The active site of GR is calculated by UM062X/6-311 G (d,p) method.

### Molecular dynamics study

#### Utilization of carbon nanotube to deliver the drug onto the protein, GR

The MD simulation of unsupported carmustine post molecular docking calculation lands the drug in the NADPH binding domain, which is a continuous and globular residue chain from residues 158–293^48^. The results of MD simulation of GR-carmustine system in the form of snapshots have been assembled and presented in the supporting information file.

**Figure 6:**
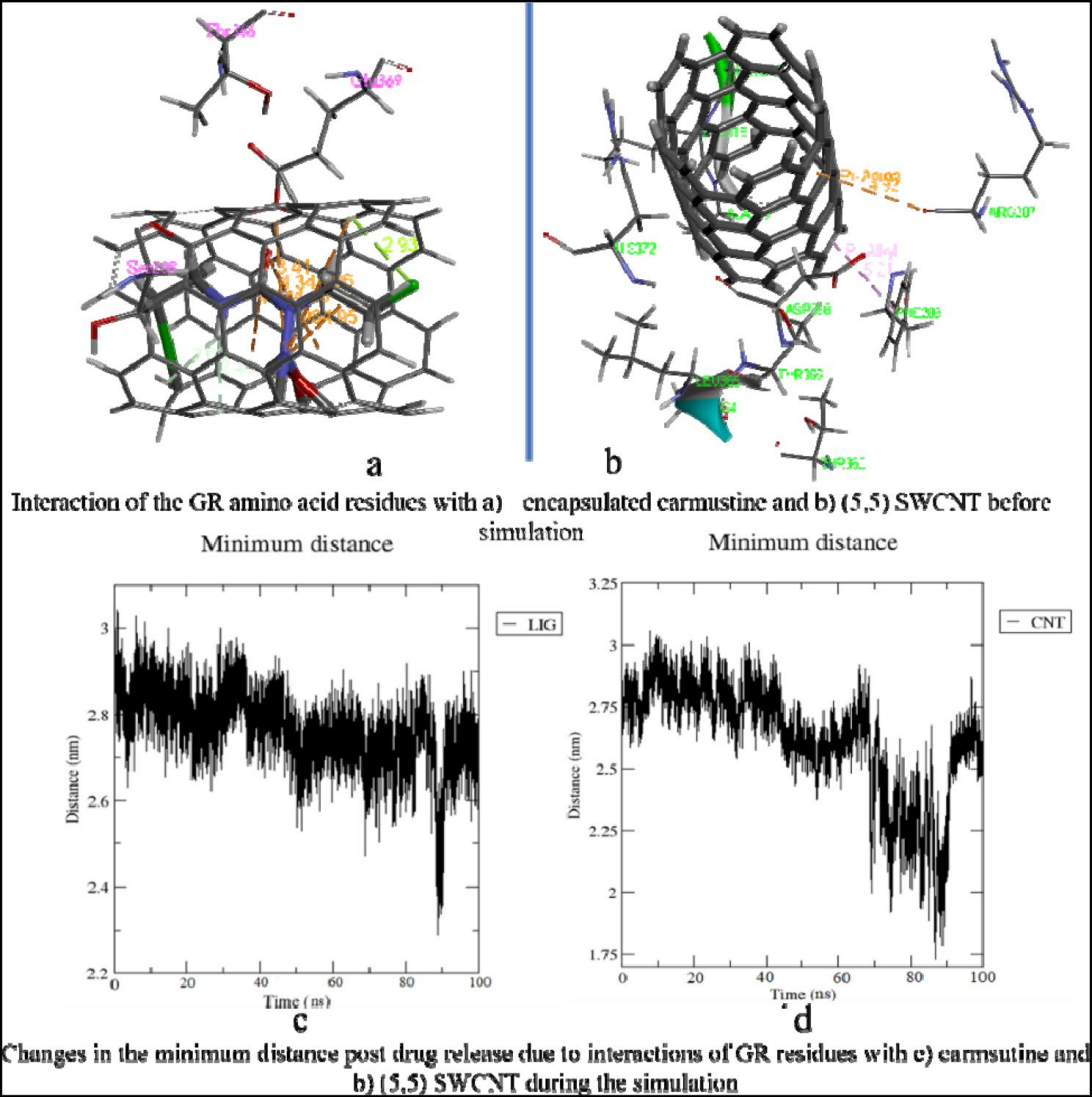
Impact of interactions of GR amino acid with the drug and the nanotube.

**Figure 7:**
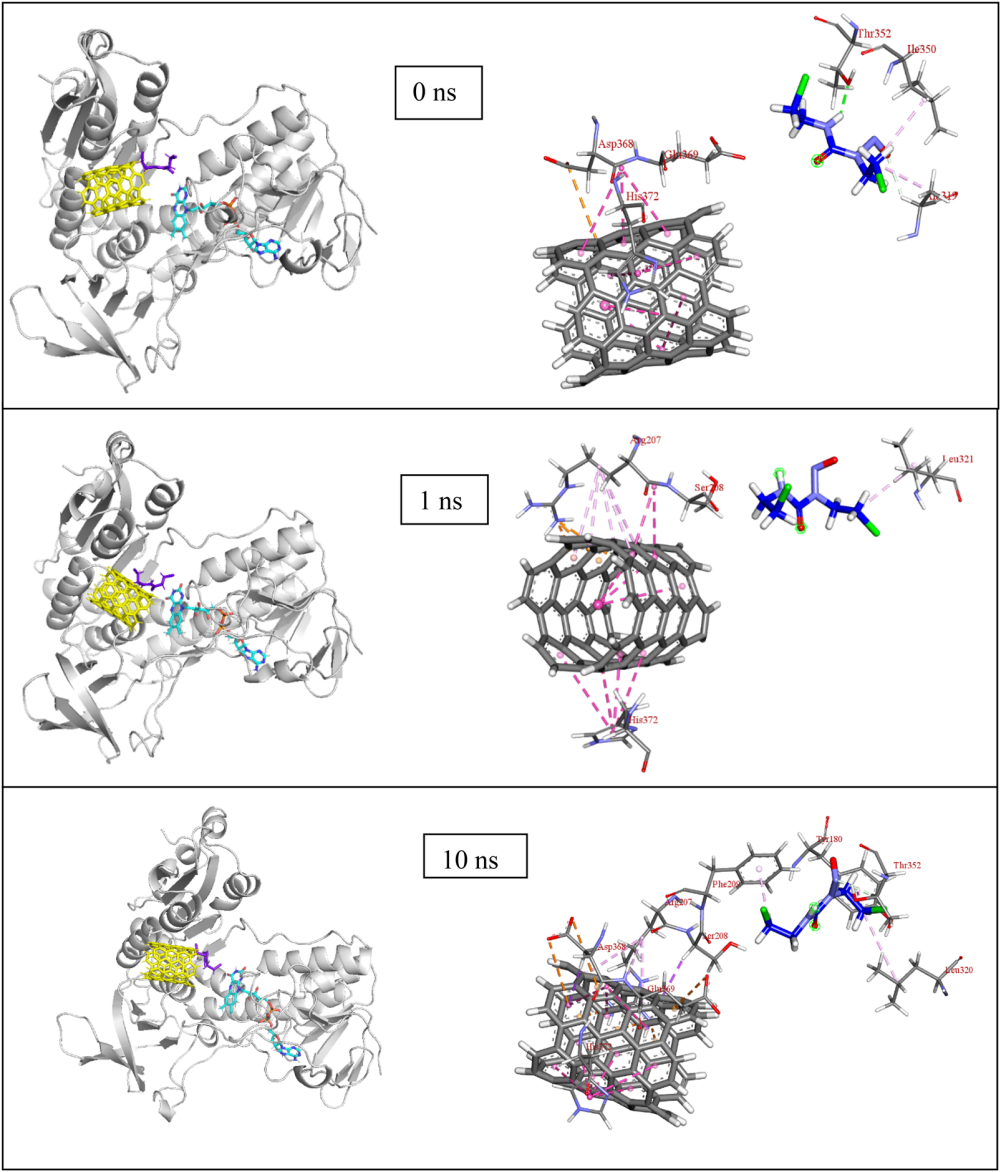

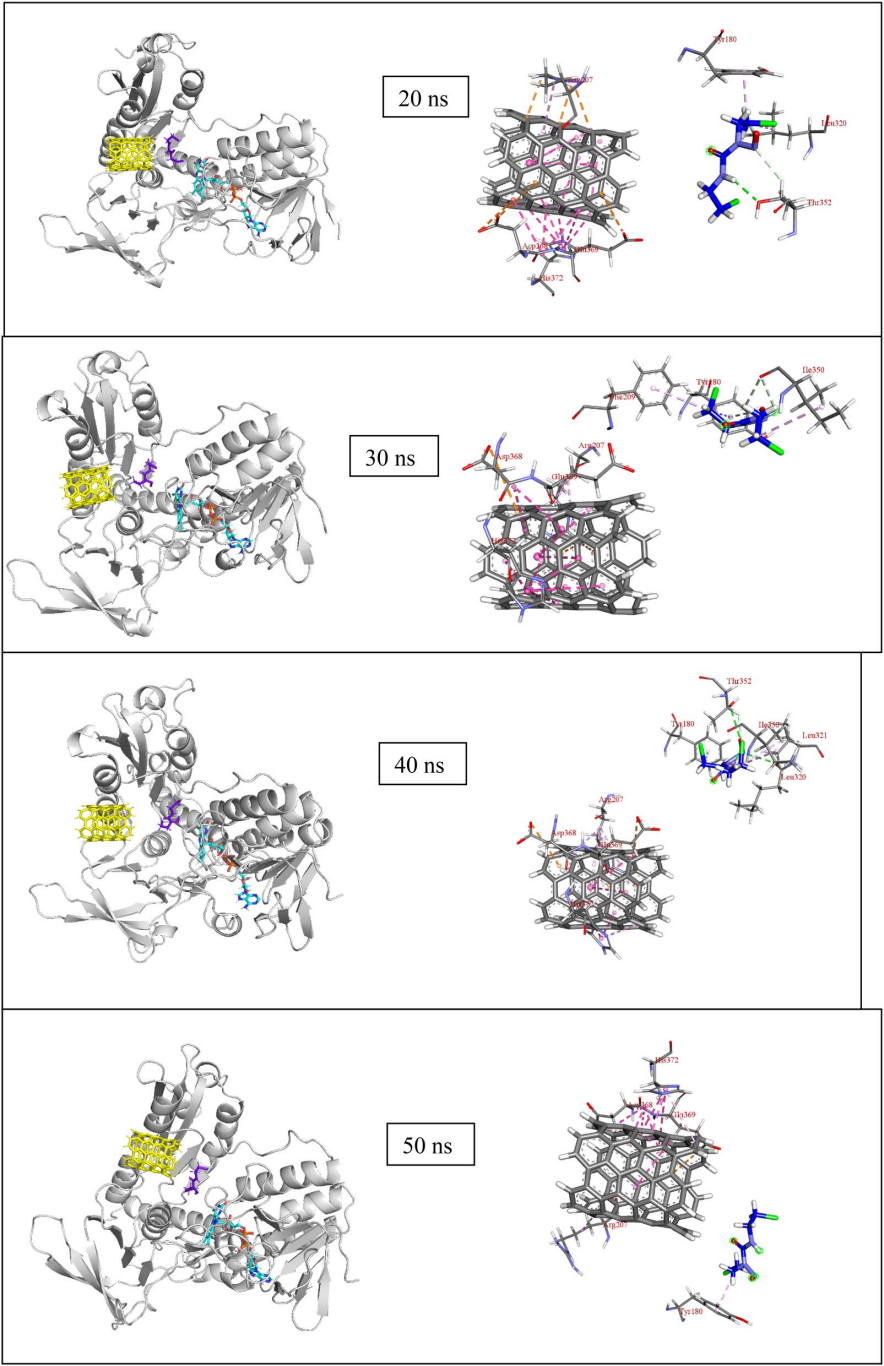

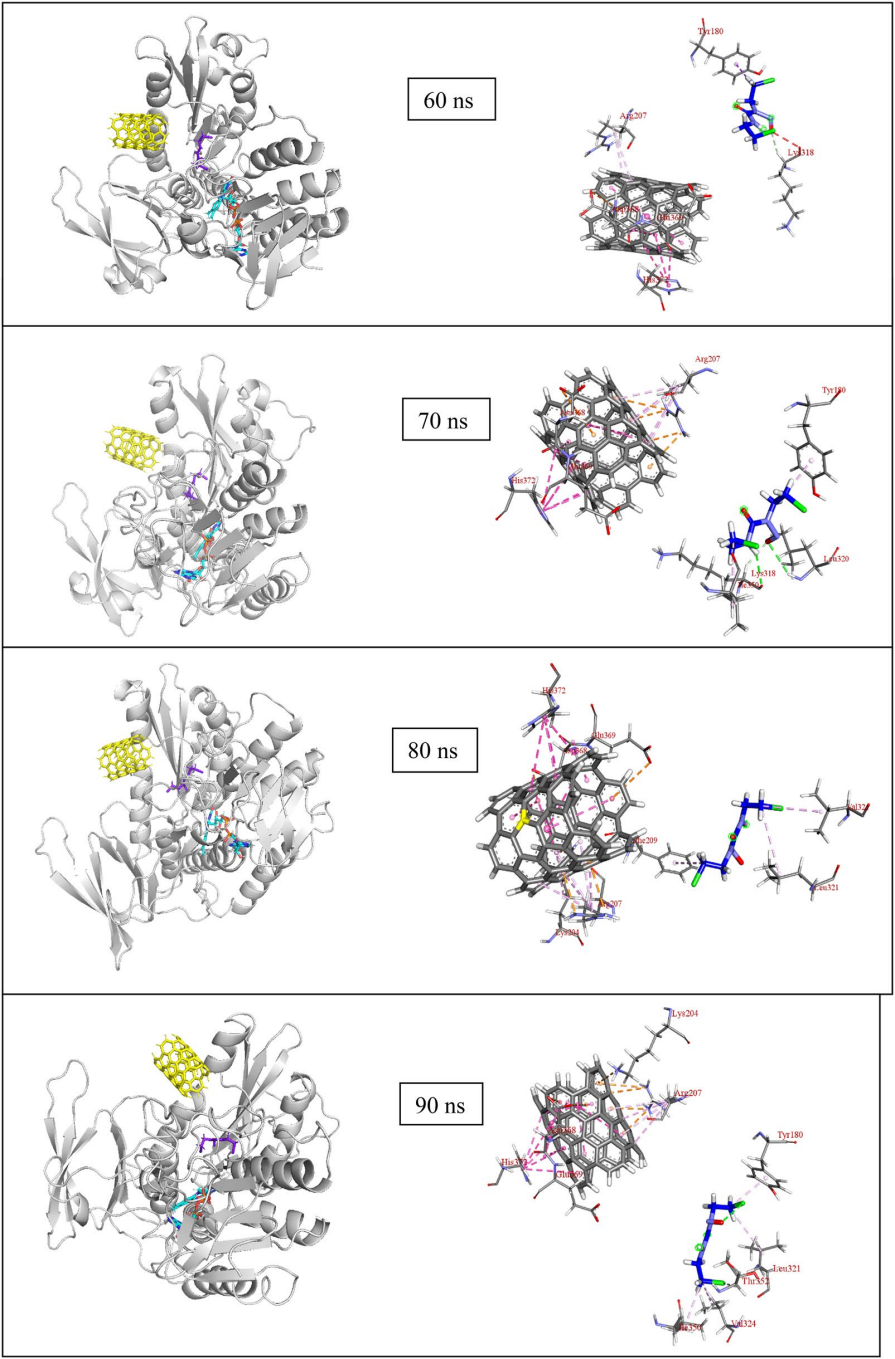

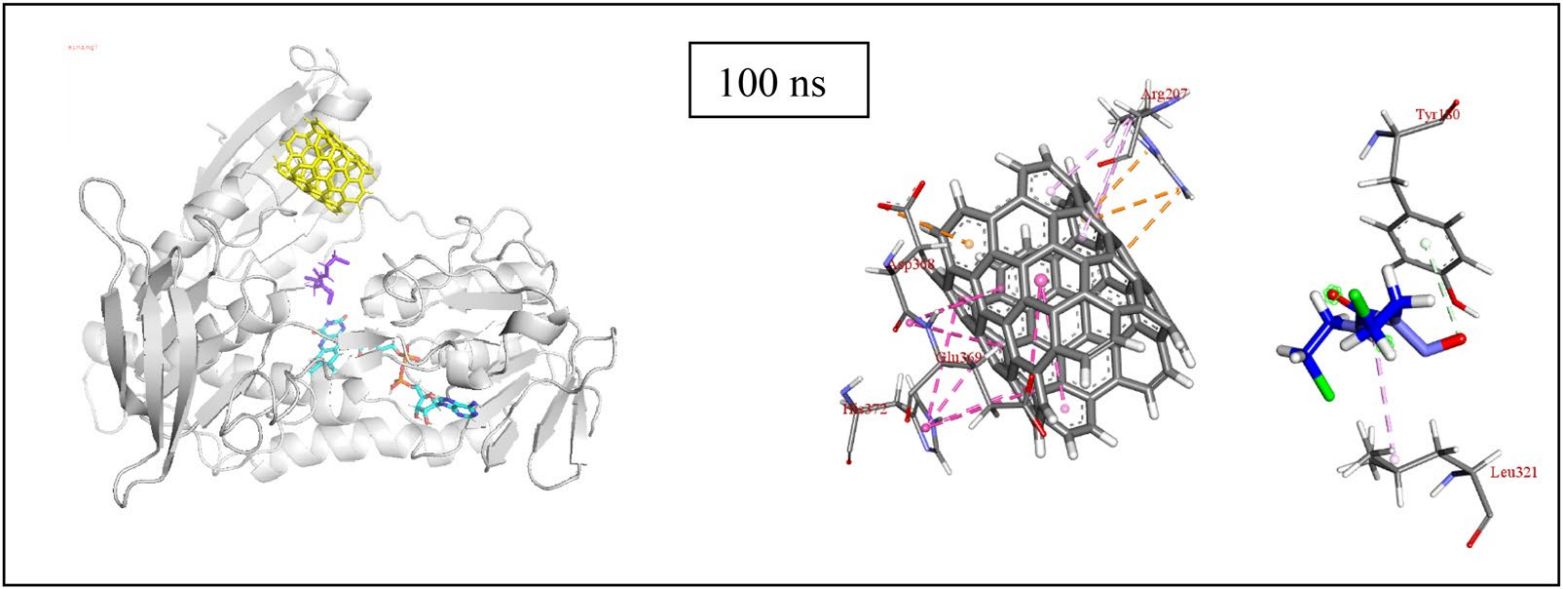
(**a**) Snapshot of MD results (shown at an interval of 10 ns) with fixed axis at each time step (**b**) Interacting atoms of GR with Carmustine and SWCNT at the binding pocket.

Before the drug release process, carmustine is found to be held inside the SWCNT by pi-anion, pi-lone pair type of interactions (Fig. 6a) and the nanotube itself interacts with the surrounding residues (Fig. 6b). The MD simulation of SWCNT encapsulated carmustine with a GR enzyme for 100 ns (Fig. 7a) illustrates that the carmustine does not remain stacked onto the nanotube sidewall, however, it is released from the cavity and is observed to interact with the adjacent residues around the binding cavity of GR. In the initial 30 ns, the fluctuation on the position of carmustine drug brings it midway between the protein and the SWCNT. Overall, the movement is not much initially, and the drug and the SWCNT remain in close proximity to the protein. It is indicated from the simulation trajectory that the free, mobile movement of the carmustine molecule is restricted in presence of the nanotube, thus preventing the drug from randomly rattling throughout the protein. The drug over time moves towards the protein away from the SWCNT endohedral cavity.

Once released, the GR amino acids that interact with the drug are Phe209, Ala319, Ile350 and Val354 at a distance of 2 to 4 Å and also with residues Tyr180, Leu321, Lys318, Leu320, and Thr352 (Thr352 forms several polar and hydrogen bonding interaction at 2.2 to 3.2 Å) that stabilizes the carmustine in the binding pocket (Fig. 7b). The nanotube, on the other hand, interacts with Lys204, Arg207, Ser208, Asp368, Glu369 and His372. The minimum paired distance between the carmustine ligand and Tyr180, Phe209, Lys318, Ala319, Leu320, Leu321, Ile350, Thr352 and Val354 grouped together (as group 1) has been determined. Similar evaluations have been done to obtain the minimum paired distance between SWCNT and the interacting ligands (Lys204, Arg207, Ser208, Asp368, Glu369 and His372) collectively named as group 2. The resulting plot has been represented below in Fig. 6c,d.

The minimum paired distance of the ligand to the residues is observed at 90 ns as a sharp peak as it is closest to the residue Thr352 at 2.8 Å (y2.3 nm). The nanotube is gradually pulled closer to the residues owing to multiple interactions with group 2 residues although the distance range is 4–5 Å. The nanotube interacts with Arg207 by pi-anion interaction at 4.92 Å and with Ser208 and Phe209 (by Pi-alkyl interactions). Towards the end of the simulation, the position of the nanotube has not changed significantly although the number of bonding interaction residues has increased that aids in keeping the SWCNT in place. Owing to stronger pi-alkyl interaction between the nanotube and Arg207 the shifting of the nanotube is much less during the entire period of simulation. The interaction distance lies in the range 4–5 Å. Asp368, Glu369 and imidazole ring of His372 also show amide pi-stacking and pi-pi stacking interactions respectively with the SWCNT towards the end of the simulation.

The MD simulation of (5,5) SWCNT with GR protein is depicted in Fig. 7. It is observed that throughout the simulation time, the nanotube fluctuates around the entrance cavity of the GR protein orienting randomly in the hollow region. The nanotube stays close to the binding pocket of GR and undergoes non-covalent interactions with the amino acid residues (group 2) throughout the simulation.

**Figure 8:**
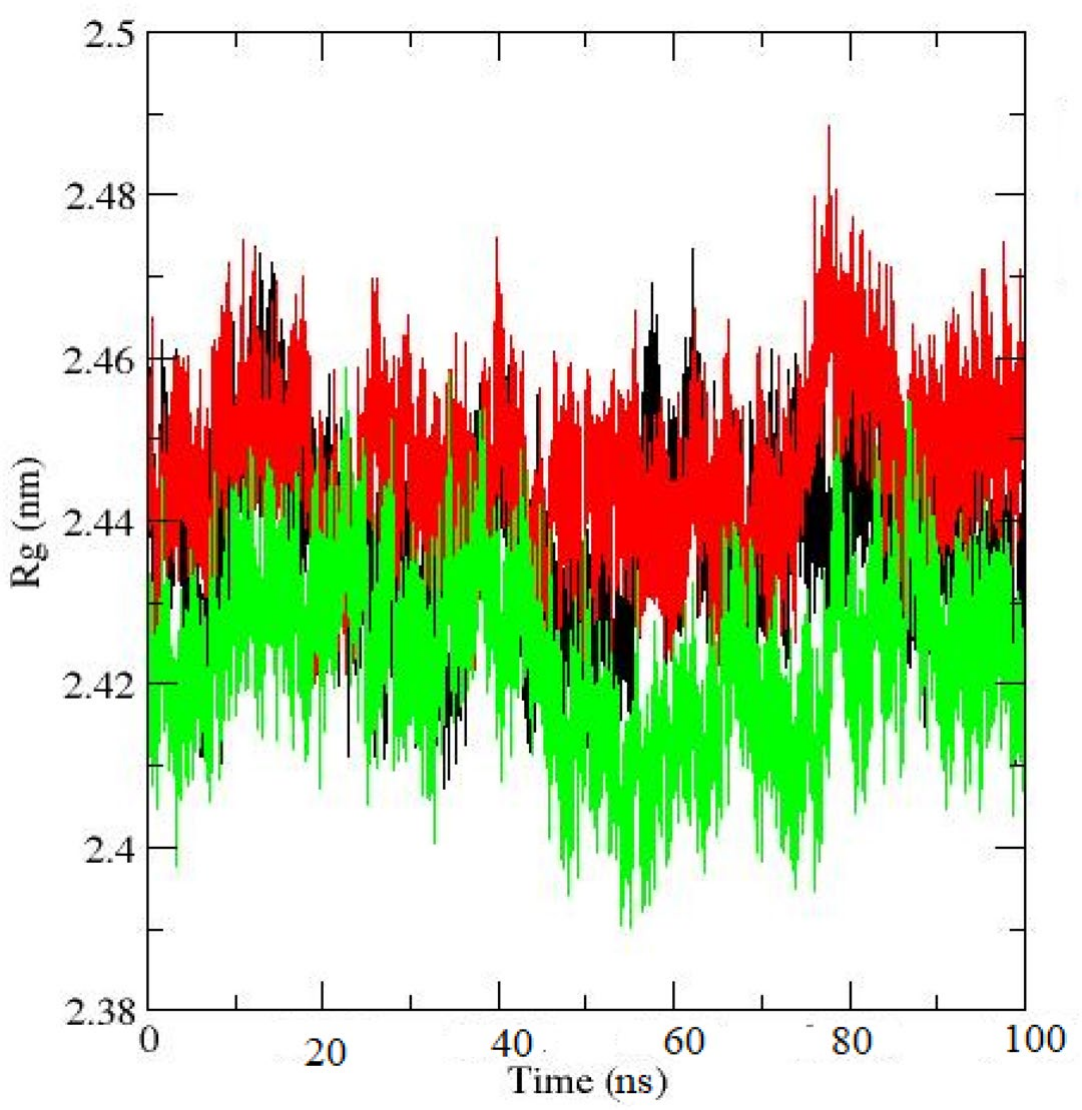
Radius of Gyration plot for protein (black = only GR; red = GR + FAD; green = GR + FAD + drug + SWCNT).

The radius of gyration, R_g_, plot (Fig. 8) which indicates the compactness of a three-dimensional protein structure, for GR protein, and for GR-SWCNT-carmustine system, shows a similar trend of change in structural features during the entirety of the simulation. The decrease in Rg value for GR (with FAD)-SWCNT-carmustine system than that of the GR protein suggests that the protein structure is more compact when the drug is bound to the active site of the GR, implying the high binding capacity of the drug to the protein.

**Figure 9:**
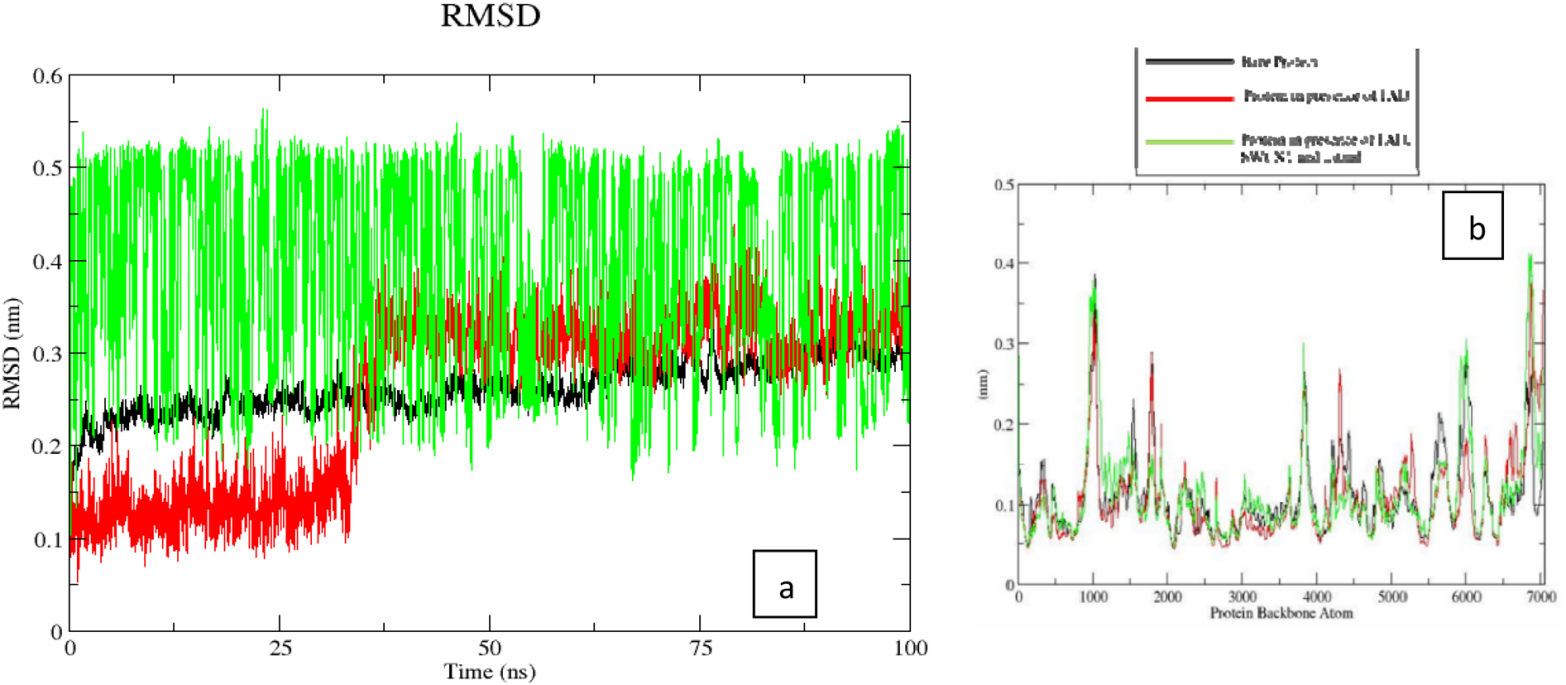
(**a**) RMSD plot (red = only SWCNT; green = only carmustine; black = GR + FAD + SWCNT + carmustine (**b**) RMS Fluctuation plot for GR.

The RMSD (Fig. 9a) for the (5,5) SWCNT shows no major change during the course of the simulation with a value of around 0.1 nm in the initial 30 ns after which it rises sharply to attain an average of 0.3 nm until the end of the simulation. The GR, on the other hand, shows a gradual increase in the RMSD from 0.2 nm with an increase in simulation time with values reaching approximately 0.3 nm at the end. The RMSD for the smaller carmustine molecule shows significant fluctuations during the course of the simulation with a maximum value of 0.55 nm at about 25 ns and a minimum of 0.2 nm at about 60 ns, the average of which (0.375 nm) is fairly consistent in the simulation.

At the beginning of the simulation i.e. 0–20 ns, the drug is in interaction with Ala319 (3.36 Å) and Thr352 (2.05 Å at 0 ns, 2.74, 2.62 Å at 10 ns and 2.96 and 2.51 Å at 20 ns). In the next 10 ns the drug moves to form non-bonded interaction with Ile350 at 3.06 and 2.93 Å (at 30 ns). The drug moves further to interact with Thr352 (2.73, 3.23 Å) and Leu320 (at 3.35 Å) at 40 ns and Val352, Tyr180, Phe209 at 4.23 Å, 4.38 Å and 5.45 Å respectively. Similar interactions continue with Leu321 (3.86 Å), Tyr180 (2.69 Å) and Lys318 (2.66 Å) at 60 ns, Leu320 (2.94 Å) and Lys318 (2.54 and 2.65 Å) at 70 ns, however, no strong interactions are observed at 80 ns. During the final nanoseconds (90 and 100 ns) the drug interacts with Thr352 at 2.87 Å and Tyr180 at 4 Å respectively. The nanotube, on the other hand, does not show such movement of getting inside the enzyme cavity as it is held by group 2 residues with multiple interactions at a range of 3–5.5 Å. It has been observed that with His372, there occurs multiple pi-pi stacked interactions at each time step. Similarly, Arg207 forms pi-alkyl interaction, Asp368 forms amide-pi interaction and Ser208 forms pi-sigma type of interactions that prove it difficult for the bulky SWCNT to move.

The RMSF (root mean square fluctuation) of the protein does not show any deformation in the secondary structure of the GR backbone (Fig. 9b) in presence of SWCNT carrying ligand except around atom numbers 1200–1500 (y0.15 nm), number 2500 (y0.125 nm), 3800 (y0.3 nm), the value decreases from atom number 4000 with slight increase at atom number 6000 and around 6900. The RMSF reaches a maximum value at around 7000 (y0.41 nm) which corroborates that, during the course of the simulation, although selected residues interact with the ligand and nanotube, overall, the protein backbone is not perturbed by the interacting ligand. The RMSF of the GR protein in the SWCNT-GR-ligand system remains more or less uniform around 0.1 nm with major fluctuations in some of the protein atoms. The RMS fluctuations suggest that overall, there is no structural deformation in the protein conformation observed and the fluctuations in the amino acid atom numbers can be accounted for from the interaction nanotube side-wall and the edges. The MD simulation of the carmustine drug with GR protein without SWCNT support shows that the carmustine drug randomly rattles around the NADPH binding cavity and interacting with Ser177, Ala199, Ile198, Ser 225, Ala288, Gly290, and Arg291, amino acid residues (Figure S2 in supplementary file). Although carmustine does enter in the binding pocket of GR as observed in the docking studies, however, under the support of SWCNT carmustine, does not enter into the same binding pocket which can be attributed to the long- distance carmustine-SWCNT interaction. Under suitable physiological conditions, the carmustine molecule can bind with the protein in a more effective manner. Although the RMSF of the protein backbone shows significant variations in most of the atoms mostly around 1000 and around 6000, overall, the GR secondary structure remains stable at around 0.15 nm throughout the course of the simulation.

In summary, we can conclude that the noncovalent functionalization of carmustine onto carbon nanotubes mediated by pi-pi stacking can modulate the properties of the nanotube. The presence of nanotube support facilitates in the loading and delivery of a carmustine drug onto a GR protein by targeting the drug towards the binding cavity without the nanotube affecting the structural conformation of GR.

## Conclusion

In this study, molecular docking, molecular dynamic simulation, QM/MM binding energy calculation and post simulation analysis has been carried out to seek detailed information of carmustine, SWCNT, GR system. Molecular docking evaluates the active site for carmustine in Glutathione reductase environment. The study is further extended to observe the release of carmustine from carbon nanotube onto the GR protein using MD simulation. In the GR-carmustine system (unsupported) the active site of the protein comprises of the residues Pro160, Ser161, Pro163, Ser177, Gly194, Gly196, Tyr197, Ala199, Ile198, Gly204, Trp287, Ala288, Ile289, Gly290 and Arg291, that play a key role in binding with the drug moiety as observed during docking and refined using QM/MM calculation. The two-layer QM/MM calculations are employed to analyze the stability and energetic details of the interacting carmustine with protein. The interaction energy is computed to be higher in the solvent phase than in the gas phase indicating higher stability of the GR-carmustine adduct in an aqueous medium.

The analysis of the MD result demonstrates the movement of the drug within the active site owing to the formation of multiple polar interactions and hydrogen bond formation. It is observed that the drug forms multiple favorable interactions preferentially with 9 amino acids (group 1) that stabilizes the drug in the binding pocket during its movement. SWCNT on the other hand does not interact with the protein binding site nor does it move as much owing to its bulkiness and several interactions with 6 amino acids (group 2) laying on the outer side of carmustine binding region. Study of the effect of drug-delivery using carbon nanotube is a nascent field. It is anticipated that such studies would bring about a change in perspective of researchers and people at large with respect to the aid that drug delivery devices provide.

## Supplementary Information

Supplementary Information.
