# Supplementary material for: Computational modelling of nanotube delivery of anti-cancer drug into glutathione reductase enzyme
Source: Sci Rep. 2021 Mar 2;11:4950. doi: 10.1038/s41598-021-84006-1 (PMC7925602; doi:10.1038/s41598-021-84006-1)
Supplement: Supplementary file 1 — Supplementary Information. [file 41598_2021_84006_MOESM1_ESM.docx]

**Computational Modelling of Nanotube delivery of anti-cancer drug into Glutathione reductase enzyme**

Saheen Shehnaz Begum, Dharitri Das, Nand Kishor Gour and Ramesh Chandra Deka*

Department of Chemical Sciences, Tezpur University, Tezpur, Assam - 784028, India

*Corresponding Author: ramesh@tezu.ernet.in

Telephone: +91-9435380221, FAX: +91- 3712-267005

Coordinates

**S.A. Gas phase:**

**S.A.1. Carmsutine:**

C -2.18347 0.74245 2.8552

H -1.4957 1.08007 3.60212

H -3.18335 0.82735 3.22657

C -1.88667 -0.72913 2.51171

H -2.57443 -1.06675 1.76479

H -0.88678 -0.81403 2.14034

H -1.38939 -1.2378 4.41824

C -3.40584 -1.4367 4.23039

C -5.47345 -2.54666 4.73573

H -5.8794 -1.62542 5.09823

H -5.679 -2.63993 3.68981

C -6.11642 -3.7239 5.49222

H -5.91087 -3.63063 6.53814

H -5.71047 -4.64514 5.12972

N -2.03216 -1.55333 3.72019

N -4.01959 -2.56042 4.95251

Cl -7.8571 -3.70742 5.23268

Cl -2.00927 1.72925 1.40832

N -3.75064 -2.43838 6.321

O -3.65987 -3.41787 7.00269

O -4.05637 -0.37487 4.04899

**S.A.2. Glutathione reductase (GR) residues without carmustine:**

N-N_3 -1 31.58142 51.37514 12.72711 L

C-C_3 -1 32.54281 51.33824 11.62537 L

C-C_R -1 32.50054 50.03236 10.86106 L

O-O_R -1 31.70258 49.12582 11.22653 L

N-N_R -1 33.3443 49.80332 9.81221 L

C-C_3 -1 33.3707 48.5535 9.05845 L

C-C_R -1 33.78272 47.4026 9.94295 L

O-O_R -1 34.83842 47.49823 10.62817 L

C-C_3 -1 34.38424 48.76262 7.93141 L

C-C_3 -1 35.1039 50.07292 8.23864 L

C-C_3 -1 34.24337 50.80461 9.25747 L

N-N_R -1 33.05245 46.25669 9.95129 L

C-C_3 -1 33.44168 45.05028 10.69462 L

C-C_R -1 33.99837 44.01057 9.7473 L

O-O_R -1 34.04955 44.26189 8.51142 L

C-C_3 -1 32.23334 44.5193 11.48103 L

O-O_3 -1 31.19905 44.15291 10.60963 L

N-N_R -1 34.51556 42.84933 10.23309 L

C-C_3 -1 35.09338 41.79335 9.3859 L

C-C_R -1 34.30839 40.50725 9.57376 L

O-O_R -1 34.02021 40.14926 10.74982 L

C-C_3 -1 36.58203 41.57325 9.73215 L

O-O_3 -1 36.71523 41.16164 11.06905 L

C-C_3 -1 37.40092 42.84924 9.50369 L

N-N_R -1 33.92032 39.72407 8.52155 L

C-C_3 -1 33.14644 38.49662 8.68134 L

C-C_R -1 33.98658 37.42659 9.32489 L

O-O_R -1 35.08575 37.08522 8.80711 L

C-C_3 -1 32.72147 38.09618 7.26794 L

C-C_3 -1 33.68343 38.81743 6.33096 L

C-C_3 -1 34.22864 39.99754 7.12464 L

N-N_3 -1 39.02681 45.21378 7.10201 L

C-C_3 -1 38.18736 46.41819 7.11302 L

C-C_R -1 38.34465 47.22859 5.83808 L

O-O_R -1 39.24851 46.93015 5.00888 L

C-C_3 -1 38.48673 47.27687 8.36245 L

O-O_3 -1 39.83425 47.67424 8.36117 L

C-C_3 -1 38.169 46.51054 9.65136 L

N-N_R -1 37.48281 48.24766 5.575 L

C-C_3 -1 37.48423 49.03001 4.33238 L

C-C_R -1 37.92166 50.43914 4.6229 L

O-O_R -1 38.9358 50.91103 4.03792 L

C-C_3 -1 36.0887 49.00756 3.69081 L

O-O_3 -1 35.12506 49.49175 4.58598 L

N-N_3 -1 30.2176 39.51683 0.01214 L

C-C_3 -1 30.39765 40.14237 1.31747 L

C-C_R -1 31.04947 41.48503 1.15721 L

O-O_R -1 32.20485 41.54436 0.65427 L

N-N_R -1 30.41269 42.61455 1.56695 L

C-C_3 -1 31.01677 43.94736 1.5168 L

C-C_R -1 30.12851 44.89253 0.76419 L

O-O_R -1 30.52357 45.3591 -0.33914 L

N-N_R -1 28.93556 45.27538 1.29257 L

C-C_3 -1 28.00705 46.19148 0.62652 L

C-C_R -1 27.8388 47.43552 1.44717 L

O-O_R -1 26.70756 47.69994 1.93856 L

N-N_R -1 28.89435 48.26863 1.6452 L

C-C_3 -1 28.8262 49.49404 2.44845 L

C-C_R -1 29.2422 50.69371 1.65143 L

O-O_R -1 29.54896 50.58539 0.43197 L

N-N_3 0 30.7578 53.99029 5.11271 H

C-C_3 0 31.4224 52.84363 5.73616 H

C-C_R 0 32.71161 52.53842 4.98112 H

O-O_R 0 33.6482 51.98881 5.49953 H

C-C_3 0 30.53291 51.5756 5.6783 H

C-C_3 0 29.08981 51.88836 6.10335 H

C-C_3 0 31.14511 50.45894 6.52475 H

C-C_3 0 28.21336 50.6467 6.25073 H

N-N_R 0 32.69313 52.91362 3.65504 H

C-C_3 0 33.62847 52.32734 2.70653 H

C-C_R 0 34.34674 53.39631 1.87245 H

O-O_R 0 34.79521 53.18051 0.78951 H

C-C_3 0 32.96114 51.29673 1.80909 H

C-C_3 -1 35.5904 44.36958 4.33922 L

C-C_R 0 34.63573 45.49037 4.6657 L H-H_ 87 0. 0.

O-O_R -1 34.87396 46.23647 5.65435 L

C-C_3 -1 35.61286 43.36756 5.50837 L

C-C_R -1 36.32322 42.0909 5.14932 L

C-C_R -1 37.49205 41.67861 5.64916 L

C-C_R -1 35.89271 41.13269 4.33098 L

N-N_R -1 37.81341 40.46666 5.14797 L

C-C_R -1 36.78981 40.16134 4.33549 L

C-C_R -1 34.71201 41.03216 3.58595 L

C-C_R -1 36.6193 38.98308 3.59863 L

C-C_R -1 34.5191 39.85868 2.83452 L

C-C_R -1 35.49756 38.83907 2.85852 L

N-N_R 0 33.48377 45.63778 3.95768 H

C-C_3 0 32.52574 46.71868 4.12569 H

C-C_R 0 31.14779 46.20091 4.59443 H

O-O_R 0 30.11378 46.80499 4.38249 H

C-C_3 0 32.34681 47.51535 2.83447 H

N-N_R 0 31.18768 45.02841 5.27405 H

C-C_3 0 29.9879 44.26323 5.53742 H

C-C_R 0 28.95762 44.92026 6.46802 H

O-O_R 0 27.87997 44.40881 6.54471 H

C-C_3 0 30.3542 42.87738 6.09846 H

C-C_3 0 31.24215 42.98347 7.35157 H

C-C_3 0 31.00769 42.00081 5.03316 H

C-C_3 0 31.07842 41.78745 8.28506 H

N-N_R 0 29.26601 46.11267 7.23765 L H-H_ 94 0. 0.

C-C_3 -1 28.20428 46.94614 7.79799 L

C-C_R -1 28.69431 47.69583 9.00293 L

O-O_R -1 29.66445 48.49096 8.87388 L

N-N_R -1 28.05854 47.57126 10.19902 L

C-C_3 -1 28.34113 48.43451 11.35586 L

C-C_R -1 29.06975 47.64794 12.42905 L

O-O_R -1 29.19017 46.39858 12.31687 L

C-C_3 -1 27.03035 49.0262 11.92199 L

C-C_3 -1 26.0872 49.63333 10.8632 L

C-C_3 -1 26.72266 50.72555 9.99302 L

N-N_R -1 27.26249 51.811 10.81327 L

C-C_R -1 28.57824 52.35648 10.63293 L

N-N_R -1 29.37033 51.87639 9.7267 L

N-N_R -1 29.00933 53.44541 11.45267 L

H-H_ -1 32.33015 52.17949 10.9331 L

H-H_ -1 31.8036 50.60296 13.39709 L

H-H_ -1 35.11008 47.92376 7.85162 L

H-H_ -1 33.85475 48.84439 6.95936 L

H-H_ -1 36.09845 49.85455 8.68402 L

H-H_ -1 35.245 50.68054 7.32012 L

H-H_ -1 34.88357 51.24838 10.05002 L

H-H_ -1 33.64938 51.60023 8.7563 L

H-H_ -1 32.36765 48.36975 8.61393 L

H-H_ -1 31.87476 45.31704 12.16948 L

H-H_ -1 32.53253 43.64428 12.0992 L

H-H_ -1 30.45909 43.82241 11.18211 L

H-H_ -1 34.2377 45.28911 11.43365 L

H-H_ -1 32.2101 46.19485 9.33382 L

H-H_ -1 36.97792 40.78137 9.05262 L

H-H_ -1 38.48156 42.63391 9.6407 L

H-H_ -1 37.10356 43.63597 10.22863 L

H-H_ -1 37.24871 43.23239 8.47406 L

H-H_ -1 37.61466 40.74986 11.1546 L

H-H_ -1 35.05387 42.10003 8.32175 L

H-H_ -1 34.52499 42.70083 11.26789 L

H-H_ -1 32.74088 36.99623 7.10148 L

H-H_ -1 31.68797 38.46046 7.07714 L

H-H_ -1 34.51707 38.13499 6.05442 L

H-H_ -1 33.16643 39.1534 5.40654 L

H-H_ -1 35.32705 40.07897 6.98585 L

H-H_ -1 33.72358 40.93246 6.80091 L

H-H_ -1 32.24239 38.69151 9.30078 L

O-O_R -1 33.55341 36.8321 10.50895 L

H-H_ -1 37.833 48.1777 8.34089 L

H-H_ -1 38.30733 47.17638 10.52929 L

H-H_ -1 38.84238 45.63448 9.76297 L

H-H_ -1 37.11439 46.16149 9.64157 L

H-H_ -1 39.91023 48.43678 8.99267 L

H-H_ -1 37.12568 46.09045 7.16639 L

H-H_ -1 40.03025 45.47956 6.97227 L

H-H_ -1 36.092 49.62953 2.7671 L

H-H_ -1 35.83775 47.96222 3.40532 L

H-H_ -1 34.25822 49.46126 4.10655 L

H-H_ -1 38.18859 48.58906 3.59227 L

H-H_ -1 36.76123 48.48488 6.28953 L

H-H_ -1 31.05191 39.50194 1.94754 L

H-H_ -1 29.41402 40.23229 1.83005 L

H-H_ -1 29.48201 40.05086 -0.50604 L

H-H_ -1 32.02305 43.92529 1.04523 L

H-H_ -1 31.14553 44.31859 2.55097 L

H-H_ -1 29.46202 42.53408 1.99432 L

H-H_ -1 28.35888 46.47487 -0.38842 L

H-H_ -1 28.64959 44.8962 2.22411 L

H-H_ -1 29.50907 49.39013 3.31565 L

H-H_ -1 27.80324 49.67139 2.84201 L

H-H_ -1 29.81844 48.01408 1.22803 L

H-H_ 0 30.50579 51.24514 4.63252 H

H-H_ 0 30.65889 49.5061 6.31325 H

H-H_ 0 31.02916 50.67592 7.58994 H

H-H_ 0 32.21022 50.35885 6.32118 H

H-H_ 0 29.10777 52.43759 7.05395 H

H-H_ 0 28.63084 52.54572 5.35888 H

H-H_ 0 27.17206 50.92756 6.41914 H

H-H_ 0 28.5331 50.03267 7.09439 H

H-H_ 0 28.25437 50.02286 5.3549 H

H-H_ 0 31.71 53.01189 6.78322 H

H-H_ 0 29.85712 54.15933 5.54575 H

H-H_ 0 32.18361 51.76615 1.19557 H

H-H_ 0 32.50588 50.52174 2.41976 H

H-H_ 0 33.695 50.85699 1.13497 H

H-H_ 0 34.40517 51.86117 3.31711 H

H-H_ 0 31.79594 53.24927 3.31901 H

H-H_ 0 34.00459 54.55624 3.3038 H

H-H_ -1 36.10495 43.83894 6.38654 L

H-H_ -1 34.5732 43.11322 5.80995 L

H-H_ -1 38.10008 42.2163 6.3638 L

H-H_ -1 33.96598 41.81454 3.58972 L

H-H_ -1 33.61994 39.73203 2.24746 L

H-H_ -1 37.36667 38.20045 3.62613 L

H-H_ -1 35.34364 37.93483 2.28398 L

H-H_ -1 35.24627 43.84934 3.41773 L

H-H_ 0 31.63966 48.32765 2.98809 H

H-H_ 0 33.30976 47.91103 2.50901 H

H-H_ 0 31.95412 46.87515 2.04303 H

H-H_ 0 32.84745 47.41142 4.90784 H

H-H_ 0 33.36766 45.09687 3.12067 H

H-H_ 0 29.4 42.42239 6.38347 H

H-H_ 0 31.35452 41.06086 5.46332 H

H-H_ 0 31.87261 42.49782 4.58536 H

H-H_ 0 30.30383 41.76409 4.23612 H

H-H_ 0 31.01732 43.90427 7.89901 H

H-H_ 0 32.28899 43.06349 7.03699 H

H-H_ 0 31.77233 41.84034 9.12595 H

H-H_ 0 30.06288 41.75382 8.68631 H

H-H_ 0 31.25929 40.84687 7.76311 H

H-H_ 0 29.42861 44.13064 4.60311 H

H-H_ 0 32.07897 44.55476 5.25865 H

H-H_ -1 27.32519 46.32245 8.0673 L

H-H_ -1 27.87583 47.67428 7.02768 L

H-H_ -1 30.237 46.49681 7.20592 L

H-H_ -1 27.27116 49.8068 12.67582 L

H-H_ -1 26.46912 48.22476 12.45159 L

H-H_ -1 25.20626 50.0645 11.38676 L

H-H_ -1 25.70284 48.82789 10.201 L

H-H_ -1 27.50107 50.27929 9.3435 L

H-H_ -1 25.94094 51.14294 9.32326 L

H-H_ -1 26.62679 52.26508 11.50733 L

H-H_ -1 30.32664 52.26277 9.56708 L

H-H_ -1 29.9636 53.85504 11.34157 L

H-H_ -1 28.37926 53.83503 12.18809 L

H-H_ -1 28.99602 49.28124 11.05788 L

H-H_ -1 27.29537 46.86292 10.29805 L

H-H_ -1 29.44646 49.22218 13.44991 L

H-H_ -1 38.65597 39.88976 5.3662 L

H-H_ -1 27.02751 45.68017 0.50675 L

H-H_ -1 29.83313 38.55718 0.16961 L

H-H_ 0 31.30768 54.83098 5.25671 H

O-O_R 0 34.47797 54.58976 2.46437 H

O-O_R -1 29.28212 51.93969 2.27485 L

H-H_ -1 29.58649 52.67246 1.6442 L

H-H_ -1 34.20819 36.13598 10.84672 L

H-H_ -1 33.56231 51.48438 12.04112 L

H-H_ -1 30.62134 51.20799 12.34887 L

O-O_R -1 29.57619 48.21713 13.44071 L

O-O_R -1 37.25461 51.17057 5.49584 L

H-H_ -1 37.6466 52.09909 5.60963 L

H-H_ -1 38.75021 44.61747 6.28893 L

N-N_3 -1 36.92708 44.91487 4.10046 L

H-H_ -1 37.55233 44.13934 3.78252 L

H-H_ -1 36.87159 45.58296 3.29717 L

**S.A.3. Glutathione reductase residues with carmustine drug:**

N-N_3 -1 26.90289 47.93123 15.24414 L

C-C_3 -1 28.24154 48.51377 15.15355 L

C-C_R -1 29.04229 47.95079 14.00005 L

O-O_R -1 28.53427 47.05639 13.26874 L

N-N_R -1 30.301 48.40408 13.73105 L

C-C_3 -1 31.09951 47.92983 12.60661 L

C-C_R -1 31.53325 46.50219 12.84876 L

O-O_R -1 32.23266 46.23894 13.86605 L

C-C_3 -1 32.29083 48.88603 12.52752 L

C-C_3 -1 32.37336 49.55988 13.89325 L

C-C_3 -1 30.98293 49.43451 14.50299 L

N-N_R -1 31.10733 45.50501 12.02456 L

C-C_3 -1 31.33211 44.07644 12.2949 L

C-C_R -1 32.19426 43.4445 11.22667 L

O-O_R -1 32.31843 44.00506 10.10294 L

C-C_3 -1 29.9851 43.34678 12.41963 L

O-O_3 -1 29.22378 43.47545 11.24792 L

N-N_R -1 32.84612 42.28043 11.49488 L

C-C_3 -1 33.76711 41.6104 10.56097 L

C-C_R -1 33.30481 40.18369 10.3166 L

O-O_R -1 32.66024 39.59429 11.22873 L

C-C_3 -1 35.20118 41.62208 11.13818 L

O-O_3 -1 35.23677 40.91662 12.35306 L

C-C_3 -1 35.69828 43.05532 11.36031 L

N-N_R -1 33.58514 39.51311 9.15803 L

C-C_3 -1 33.14232 38.14536 8.90306 L

C-C_R -1 33.91887 37.17715 9.75371 L

O-O_R -1 35.17969 37.15615 9.70183 L

C-C_3 -1 33.39341 37.9067 7.41407 L

C-C_3 -1 34.42715 38.94794 7.00411 L

C-C_3 -1 34.30184 40.07553 8.0207 L

N-N_3 -1 37.69567 46.12452 9.77795 L

C-C_3 -1 36.57515 47.04404 9.54439 L

C-C_R -1 36.92437 48.10801 8.51992 L

O-O_R -1 38.1273 48.28221 8.17909 L

C-C_3 -1 36.11322 47.68935 10.87101 L

O-O_3 -1 37.16364 48.43354 11.43381 L

C-C_3 -1 35.6341 46.62923 11.87038 L

N-N_R -1 35.93657 48.85776 7.96024 L

C-C_3 -1 36.17208 49.93029 6.98393 L

C-C_R -1 35.65052 51.23277 7.52697 L

O-O_R -1 36.43022 52.21897 7.6407 L

C-C_3 -1 35.51177 49.58557 5.64207 L

O-O_3 -1 34.12915 49.42339 5.79386 L

N-N_3 -1 34.11692 38.80969 0.25975 L

C-C_3 -1 33.57259 39.36945 1.49184 L

C-C_R -1 33.7989 40.85316 1.52521 L

O-O_R -1 34.98226 41.28961 1.50701 L

N-N_R -1 32.74517 41.71046 1.58004 L

C-C_3 -1 32.88777 43.16352 1.68967 L

C-C_R -1 32.2285 43.8337 0.52251 L

O-O_R -1 32.94062 44.45977 -0.30942 L

N-N_R -1 30.87607 43.79845 0.39116 L

C-C_3 -1 30.15041 44.4224 -0.71673 L

C-C_R -1 29.28998 45.53391 -0.19684 L

O-O_R -1 28.03571 45.43526 -0.28779 L

N-N_R -1 29.86254 46.63666 0.3542 L

C-C_3 -1 29.09887 47.74988 0.92503 L

C-C_R -1 29.36068 49.02734 0.18784 L

O-O_R -1 30.12292 49.04962 -0.81786 L

N-N_3 0 30.09689 54.31569 4.33186 H

C-C_3 0 30.92576 53.6861 5.35953 H

C-C_R 0 32.07984 52.93671 4.69205 H

O-O_R 0 33.11985 52.69786 5.30511 H

C-C_3 0 30.06103 52.72181 6.19853 H

C-C_3 0 29.14238 53.50191 7.15617 H

C-C_3 0 30.90636 51.72901 6.99501 H

C-C_3 0 27.92046 52.69906 7.6044 H

N-N_R 0 31.87043 52.54684 3.42687 H

C-C_3 0 32.80278 51.70087 2.68178 H

C-C_R 0 34.02646 52.52003 2.22702 H

O-O_R 0 34.3092 52.67756 1.07529 H

C-C_3 0 32.09618 51.06059 1.5014 H

C-C_3 -1 35.69968 44.75614 5.94657 L

C-C_R 0 34.45143 45.58339 5.77551 L H-H_ 87 0. 0.

O-O_R -1 34.08899 46.35039 6.70845 L

C-C_3 -1 35.47528 43.72735 7.07058 L

C-C_R -1 36.58128 42.70902 7.13477 L

C-C_R -1 37.48702 42.59918 8.11111 L

C-C_R -1 36.82096 41.72306 6.27233 L

N-N_R -1 38.30667 41.55308 7.87397 L

C-C_R -1 37.85575 41.03313 6.72211 L

C-C_R -1 36.15159 41.35352 5.09966 L

C-C_R -1 38.34725 39.9034 6.05733 L

C-C_R -1 36.62923 40.22216 4.41257 L

C-C_R -1 37.73935 39.50809 4.91715 L

N-N_R 0 33.67422 45.43721 4.65738 H

C-C_3 0 32.54044 46.28123 4.32391 H

C-C_R 0 31.18791 45.52594 4.36866 H

O-O_R 0 30.1954 45.94834 3.80461 H

C-C_3 0 32.70215 46.93447 2.95096 H

N-N_R 0 31.19893 44.38069 5.10124 H

C-C_3 0 30.19349 43.3445 4.9383 H

C-C_R 0 28.76055 43.66661 5.39336 H

O-O_R 0 27.89324 42.89055 5.10477 H

C-C_3 0 30.63091 42.07412 5.69727 H

C-C_3 0 30.85386 42.35124 7.19626 H

C-C_3 0 31.85246 41.415 5.05903 H

C-C_3 0 30.63471 41.10477 8.04982 H

N-N_R 0 28.46847 44.84649 6.17798 L H-H_ 94 0. 0.

C-C_3 -1 27.17832 45.06405 6.83417 L

C-C_R -1 27.34875 45.30007 8.30943 L

O-O_R -1 28.47886 45.14174 8.84826 L

N-N_R -1 26.27844 45.68936 9.05106 L

C-C_3 -1 26.35743 46.05714 10.4733 L

C-C_R -1 25.54502 45.0829 11.30703 L

O-O_R -1 24.79865 44.24174 10.73806 L

C-C_3 -1 25.83658 47.49977 10.67576 L

C-C_3 -1 26.5124 48.54859 9.76619 L

C-C_3 -1 27.98217 48.82735 10.11661 L

N-N_R -1 28.08214 49.70616 11.2845 L

C-C_R -1 28.96232 50.83837 11.34857 L

N-N_R -1 29.81724 51.06148 10.40097 L

N-N_R -1 28.90643 51.71165 12.47862 L

H-H_ 0 31.1844 48.46083 5.69169 H

C-C_R 0 29.21626 48.52112 6.08291 H

N-N_R 0 30.47806 48.15167 6.34399 H

N-N_R 0 29.0202 49.28932 4.86584 H

O-O_R 0 28.24394 48.28788 6.75835 H

C-C_3 0 30.85233 47.41676 7.53446 H

C-C_3 0 31.81527 48.24524 8.37384 H

Cl-Cl 0 32.65538 47.18487 9.54137 H

C-C_3 0 27.64041 49.67878 4.5561 H

C-C_3 0 26.81688 48.49115 4.08386 H

Cl-Cl 0 25.20143 49.08212 3.5834 H

N-N_R 0 29.91286 49.75796 3.9951 H

O-O_R 0 31.08148 49.49505 4.17708 H

H-H_ -1 28.14302 49.61291 15.02862 L

H-H_ -1 26.98862 46.89544 15.36407 L

H-H_ -1 33.24185 48.3738 12.27203 L

H-H_ -1 32.0911 49.6593 11.75294 L

H-H_ -1 33.11034 49.01979 14.52759 L

H-H_ -1 32.68182 50.62386 13.8005 L

H-H_ -1 31.0548 49.14028 15.57244 L

H-H_ -1 30.43804 50.39796 14.41037 L

H-H_ -1 30.51136 48.00858 11.66716 L

H-H_ -1 29.41234 43.79446 13.25971 L

H-H_ -1 30.14636 42.27215 12.66761 L

H-H_ -1 29.54066 42.77518 10.62052 L

H-H_ -1 31.8615 43.94931 13.26458 L

H-H_ -1 30.5304 45.75536 11.1888 L

H-H_ -1 35.88417 41.13471 10.40668 L

H-H_ -1 36.77377 43.04336 11.63644 L

H-H_ -1 35.13001 43.54195 12.18111 L

H-H_ -1 35.58019 43.65695 10.43528 L

H-H_ -1 36.18955 40.70414 12.53496 L

H-H_ -1 33.78045 42.15049 9.59052 L

H-H_ -1 32.73198 41.86051 12.44622 L

H-H_ -1 33.74412 36.87492 7.18976 L

H-H_ -1 32.45246 38.08893 6.84918 L

H-H_ -1 35.44522 38.50357 7.06212 L

H-H_ -1 34.24668 39.30731 5.96881 L

H-H_ -1 35.30801 40.42186 8.32614 L

H-H_ -1 33.7216 40.91495 7.58387 L

H-H_ -1 32.05405 38.04722 9.11573 L

O-O_R -1 33.23714 36.32011 10.61611 L

H-H_ -1 35.25342 48.36506 10.65362 L

H-H_ -1 35.28106 47.11893 12.80213 L

H-H_ -1 36.46014 45.93585 12.13453 L

H-H_ -1 34.79079 46.04777 11.44104 L

H-H_ -1 36.7555 49.05564 12.09148 L

H-H_ -1 35.72935 46.45003 9.13338 L

H-H_ -1 38.5302 46.65718 10.11582 L

H-H_ -1 35.71422 50.39892 4.909 L

H-H_ -1 35.95737 48.64391 5.25062 L

H-H_ -1 33.78437 49.16721 4.9009 L

H-H_ -1 37.26056 50.04955 6.78635 L

H-H_ -1 34.94956 48.67065 8.24572 L

H-H_ -1 34.08521 38.91078 2.36464 L

H-H_ -1 32.48918 39.12705 1.56822 L

H-H_ -1 33.52128 39.13501 -0.53659 L

H-H_ -1 33.95541 43.46818 1.74147 L

H-H_ -1 32.4025 43.50409 2.62649 L

H-H_ -1 31.77463 41.32212 1.58437 L

H-H_ -1 30.83859 44.8289 -1.4884 L

H-H_ -1 30.30975 43.30117 1.11616 L

H-H_ -1 29.40168 47.8827 1.98317 L

H-H_ -1 28.00687 47.55056 0.906 L

H-H_ -1 30.9054 46.69025 0.40656 L

H-H_ 0 29.43611 52.17567 5.47796 H

H-H_ 0 30.27021 51.13835 7.65646 H

H-H_ 0 31.64087 52.26282 7.6032 H

H-H_ 0 31.46094 51.05077 6.34777 H

H-H_ 0 29.73799 53.80124 8.02549 H

H-H_ 0 28.79768 54.43264 6.69428 H

H-H_ 0 27.40663 53.19057 8.43194 H

H-H_ 0 28.18949 51.69326 7.936 H

H-H_ 0 27.20506 52.59745 6.78438 H

H-H_ 0 31.40206 54.39829 6.04651 H

H-H_ 0 29.17025 54.5195 4.68526 H

H-H_ 0 31.54935 51.81791 0.93551 H

H-H_ 0 31.40945 50.28601 1.84174 H

H-H_ 0 32.82744 50.62173 0.82488 H

H-H_ 0 33.15693 50.93464 3.37718 H

H-H_ 0 30.99735 52.8414 3.00503 H

H-H_ 0 34.35999 52.83052 4.08548 H

H-H_ -1 35.39412 44.25678 8.04432 L

H-H_ -1 34.51483 43.19094 6.90794 L

H-H_ -1 37.56253 43.2388 8.97922 L

H-H_ -1 35.28885 41.89713 4.73762 L

H-H_ -1 36.14173 39.8905 3.50697 L

H-H_ -1 39.19255 39.35375 6.45128 L

H-H_ -1 38.10008 38.63489 4.38905 L

H-H_ -1 35.91703 44.21848 4.99688 L

H-H_ 0 31.82269 47.52159 2.69875 H

H-H_ 0 33.58399 47.57554 2.951 H

H-H_ 0 32.83292 46.1678 2.18365 H

H-H_ 0 32.43997 47.08644 5.05607 H

H-H_ 0 34.04875 44.9145 3.88498 H

H-H_ 0 29.78651 41.3857 5.59833 H

H-H_ 0 32.12755 40.51539 5.61037 H

H-H_ 0 32.72533 42.07555 5.05468 H

H-H_ 0 31.64127 41.11887 4.03144 H

H-H_ 0 30.17562 43.13625 7.54505 H

H-H_ 0 31.86666 42.7399 7.35149 H

H-H_ 0 30.8965 41.28614 9.09249 H

H-H_ 0 29.58636 40.80042 8.01314 H

H-H_ 0 31.23391 40.26458 7.69524 H

H-H_ 0 30.08384 43.10641 3.87246 H

H-H_ 0 32.12631 44.08456 5.37565 H

H-H_ -1 26.49942 44.19754 6.68793 L

H-H_ -1 26.69759 45.95488 6.38293 L

H-H_ -1 29.21023 45.56423 6.30977 L

H-H_ -1 25.95446 47.79916 11.738 L

H-H_ -1 24.74616 47.51604 10.45592 L

H-H_ -1 25.94316 49.50135 9.83494 L

H-H_ -1 26.44224 48.2235 8.70746 L

H-H_ -1 28.53022 47.88532 10.31277 L

H-H_ -1 28.46166 49.30367 9.23578 L

H-H_ -1 27.5033 49.49021 12.12587 L

H-H_ -1 30.4729 51.87384 10.42863 L

H-H_ -1 29.55373 52.52712 12.55913 L

H-H_ -1 28.2203 51.53909 13.2461 L

H-H_ -1 27.40528 45.99773 10.83589 L

H-H_ -1 25.35151 45.78701 8.57543 L

H-H_ -1 26.23784 45.77288 12.95173 L

H-H_ 0 29.95118 47.18948 8.0996 H

H-H_ 0 31.32439 46.47374 7.24255 H

H-H_ 0 31.3003 49.02001 8.93684 H

H-H_ 0 32.58179 48.70441 7.75492 H

H-H_ 0 27.19111 50.11574 5.44665 H

H-H_ 0 27.70896 50.42945 3.77001 H

H-H_ 0 26.66557 47.77508 4.8835 H

H-H_ 0 27.27145 48.00748 3.22454 H

H-H_ -1 39.09736 41.21563 8.46666 L

H-H_ -1 29.51982 43.65078 -1.20898 L

H-H_ -1 34.00849 37.77068 0.30728 L

H-H_ 0 30.5011 55.19156 4.01917 H

O-O_R 0 34.76355 53.02846 3.21408 H

O-O_R -1 28.75258 50.2002 0.63232 L

H-H_ -1 29.00875 51.00061 0.06568 L

H-H_ -1 33.86933 35.72488 11.13902 L

H-H_ -1 28.78219 48.3192 16.10406 L

H-H_ -1 26.3902 48.11565 14.35096 L

O-O_R -1 25.60634 45.07836 12.57193 L

O-O_R -1 34.38847 51.3337 7.90049 L

H-H_ -1 34.15873 52.2555 8.25588 L

H-H_ -1 37.95666 45.67391 8.87131 L

N-N_3 -1 36.82982 45.63103 6.26354 L

H-H_ -1 37.70347 45.05658 6.27498 L

H-H_ -1 36.94948 46.3114 5.47793 L

**S.B. Solvent phase:**

**S.B.1. Carmustine**:

C -2.18347 0.74245 2.8552

H -1.4957 1.08007 3.60212

H -3.18335 0.82735 3.22657

C -1.88667 -0.72913 2.51171

H -2.57443 -1.06675 1.76479

H -0.88678 -0.81403 2.14034

H -1.38939 -1.2378 4.41824

C -3.40584 -1.4367 4.23039

C -5.47345 -2.54666 4.73573

H -5.8794 -1.62542 5.09823

H -5.679 -2.63993 3.68981

C -6.11642 -3.7239 5.49222

H -5.91087 -3.63063 6.53814

H -5.71047 -4.64514 5.12972

N -2.03216 -1.55333 3.72019

N -4.01959 -2.56042 4.95251

Cl -7.8571 -3.70742 5.23268

Cl -2.00927 1.72925 1.40832

N -3.75064 -2.43838 6.321

O -3.65987 -3.41787 7.00269

O -4.05637 -0.37487 4.04899

**S.B.2. Glutathione reductase (GR) residues without carmustine:**

N-N_3 30.71321 53.99144 5.1221

C-C_3 31.36112 52.84345 5.7591

C-C_R 32.67665 52.54836 5.03973

O-O_R 33.60914 51.99578 5.58015

C-C_3 30.4682 51.57944 5.66875

C-C_3 29.02199 51.90291 6.07704

C-C_3 31.06066 50.44497 6.50575

C-C_3 28.13085 50.6743 6.23405

N-N_R 32.69613 52.93239 3.71939

C-C_3 33.66743 52.37227 2.79226

C-C_R 34.3811 53.46946 1.99585

O-O_R 34.86529 53.27952 0.92108

C-C_3 33.05341 51.33227 1.86455

N-N_R 33.48077 45.63799 3.95179

C-C_3 32.52849 46.72071 4.11986

C-C_R 31.15218 46.20699 4.59712

O-O_R 30.11847 46.81236 4.38401

C-C_3 32.34479 47.5119 2.82657

N-N_R 31.18926 45.03807 5.28813

C-C_3 29.98417 44.27442 5.53399

C-C_R 28.94979 44.92572 6.46494

O-O_R 27.80045 44.42554 6.57629

C-C_3 30.33439 42.88112 6.08915

C-C_3 31.20527 42.97681 7.35306

C-C_3 30.99518 42.0037 5.02592

C-C_3 31.04022 41.76569 8.26651

H-H_ 30.4545 51.26514 4.61788

H-H_ 30.54743 49.50499 6.2981

H-H_ 30.96424 50.66402 7.57374

H-H_ 32.11969 50.3157 6.29041

H-H_ 29.03461 52.4617 7.02207

H-H_ 28.57654 52.55631 5.32178

H-H_ 27.09163 50.96994 6.38547

H-H_ 28.43295 50.07635 7.09185

H-H_ 28.17557 50.03206 5.3521

H-H_ 31.61671 53.00506 6.81528

H-H_ 29.79925 54.15237 5.52668

H-H_ 32.27647 51.78507 1.24426

H-H_ 32.61206 50.52575 2.45033

H-H_ 33.81648 50.92662 1.20199

H-H_ 34.43882 51.91507 3.41982

H-H_ 31.80671 53.26615 3.36211

H-H_ 33.97044 54.59764 3.44033

H-H_ 31.63574 48.32331 2.97118

H-H_ 33.30499 47.90853 2.49751

H-H_ 31.95357 46.86281 2.03966

H-H_ 32.85678 47.41651 4.89837

H-H_ 33.05751 44.90049 3.42553

H-H_ 29.37477 42.43147 6.36084

H-H_ 31.33365 41.0591 5.45586

H-H_ 31.86811 42.49647 4.588

H-H_ 30.29758 41.77219 4.22063

H-H_ 30.96353 43.88586 7.91188

H-H_ 32.25415 43.07288 7.05381

H-H_ 31.72346 41.81063 9.11532

H-H_ 30.02101 41.71853 8.65598

H-H_ 31.23407 40.83423 7.73195

H-H_ 29.43423 44.15277 4.59173

H-H_ 32.07584 44.55586 5.26384

H-H_ 31.25465 54.83366 5.28941

O-O_R 34.46435 54.65731 2.61189

H-H_ 33.75441 45.29241 4.8494

H-H_ 29.20672 45.80457 7.01857

**S.B.3. Glutathione reductase residues with carmustine drug:**

N 29.92177 54.23101 4.62943

C 30.70537 53.49398 5.62008

C 31.89534 52.82312 4.93536

O 32.90445 52.52005 5.57092

C 29.81019 52.43407 6.29677

C 28.82077 53.08779 7.27844

C 30.61938 51.3614 7.02532

C 27.56916 52.24002 7.50573

N 31.74705 52.56265 3.62974

C 32.70622 51.77444 2.85806

C 33.96945 52.59491 2.53636

O 34.32436 52.82059 1.41631

C 32.04781 51.25849 1.5947

N 33.67152 45.43103 4.66046

C 32.54161 46.2756 4.31823

C 31.18832 45.52196 4.35969

O 30.19849 45.94184 3.78944

C 32.71342 46.92438 2.94433

N 31.19626 44.38141 5.09926

C 30.19391 43.34226 4.94153

C 28.76323 43.65766 5.40736

O 27.82611 42.85945 5.14612

C 30.64178 42.07376 5.69707

C 30.86815 42.35286 7.19577

C 31.86559 41.42229 5.05608

C 30.636 41.11117 8.05285

H 31.19334 48.44043 5.66122

C 29.2347 48.55274 6.08733

N 30.49247 48.15992 6.33176

N 29.03535 49.31656 4.8698

O 28.2685 48.3434 6.78032

C 30.86499 47.42446 7.52217

C 31.78859 48.26425 8.39676

Cl 32.66946 47.19615 9.52815

C 27.65773 49.72137 4.57148

C 26.82299 48.54231 4.09783

Cl 25.20457 49.14371 3.62104

N 29.92314 49.74122 3.97147

O 31.08738 49.45613 4.14414

H 29.23811 51.96389 5.48406

H 29.94818 50.69423 7.57131

H 31.30693 51.82346 7.73873

H 31.22287 50.7666 6.34017

H 29.34356 53.25152 8.22684

H 28.51645 54.07944 6.92858

H 26.96753 52.63089 8.3276

H 27.81663 51.20193 7.74232

H 26.94237 52.23507 6.60982

H 31.14086 54.12668 6.4048

H 28.97077 54.37093 4.94753

H 31.75844 52.09044 0.95

H 31.17302 50.66425 1.85557

H 32.74923 50.64144 1.03534

H 33.01126 50.93937 3.49433

H 30.9003 52.91054 3.19487

H 34.20685 52.75232 4.42799

H 31.84269 47.52253 2.68777

H 33.60352 47.55406 2.94653

H 32.83692 46.15462 2.17907

H 32.4365 47.08221 5.04727

H 34.25451 45.31238 3.85669

H 29.80194 41.37961 5.60113

H 32.14716 40.52534 5.60861

H 32.73392 42.08855 5.04853

H 31.65344 41.12311 4.02957

H 30.19998 43.14715 7.543

H 31.88566 42.7297 7.34965

H 30.9014 41.29202 9.09478

H 29.58423 40.81864 8.01828

H 31.22551 40.26367 7.69892

H 30.07795 43.10681 3.87575

H 32.1202 44.09226 5.3914

H 29.95807 47.16828 8.06488

H 31.36685 46.4975 7.23036

H 31.2375 48.98953 8.99044

H 32.53518 48.78449 7.80173

H 27.21966 50.15609 5.46887

H 27.72647 50.4774 3.79036

H 26.6784 47.81942 4.89322

H 27.26656 48.06491 3.22897

H 30.32003 55.14604 4.45022

O 34.65637 53.01665 3.59748

H 34.19845 45.86031 5.39399

H 28.56426 44.55588 5.95371

**
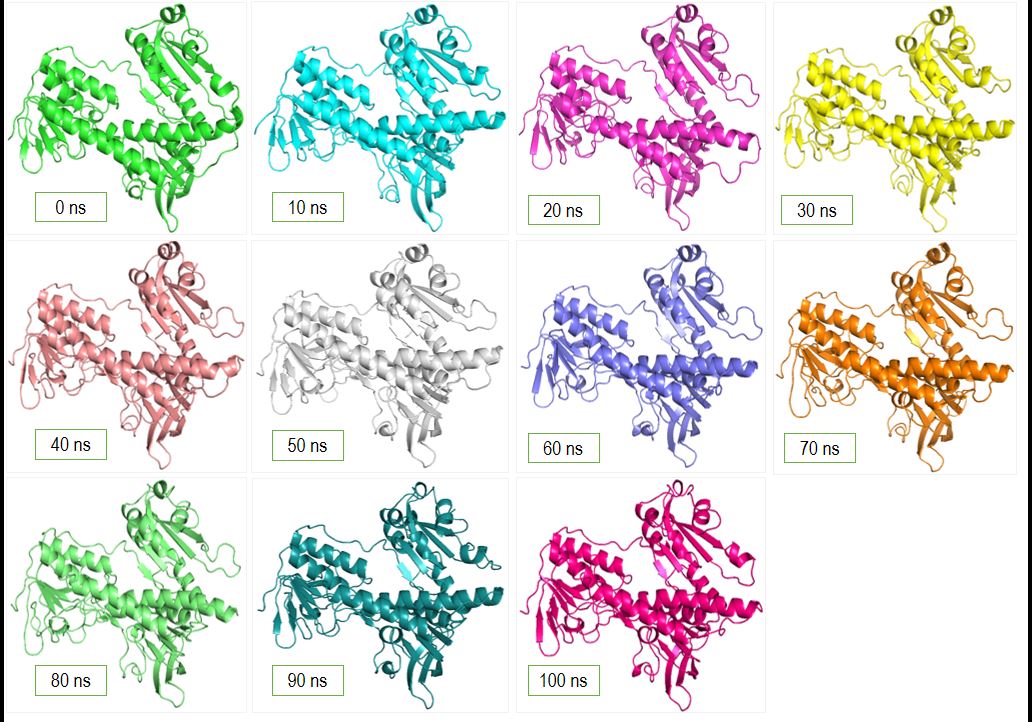
S.C.1. Figure representing the output of MD simulation**:

Figure S1: Protein movement in absence of FAD, ligand and nanotube


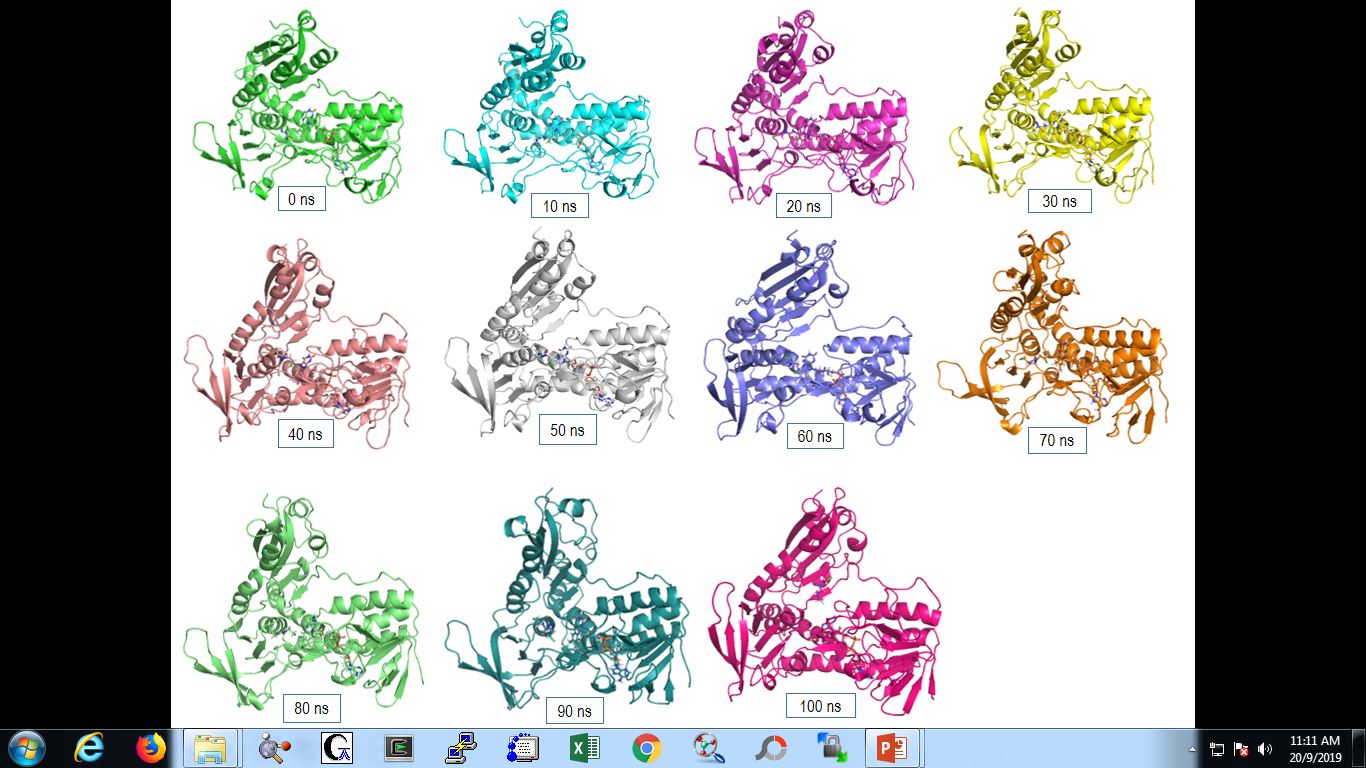


Figure S2: Trajectory of carmustine in GR absence of SWCNT


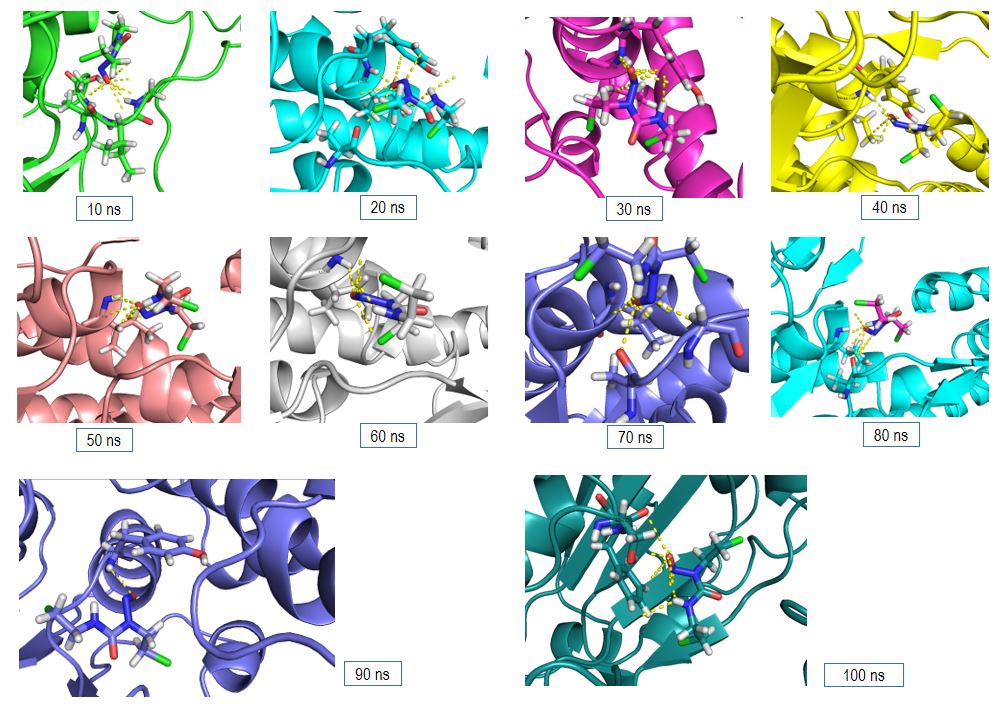


**Figure S3:** Interactions of the carmustine drug with the residues in the binding site of GR. Yellow dashed lines represent the non-covalent bonding interactions that hold the drug.
